# New Alpiniamides From *Streptomyces* sp. IB2014/011-12 Assembled by an Unusual Hybrid Non-ribosomal Peptide Synthetase *Trans*-AT Polyketide Synthase Enzyme

**DOI:** 10.3389/fmicb.2018.01959

**Published:** 2018-08-22

**Authors:** Constanze Paulus, Yuriy Rebets, Josef Zapp, Christian Rückert, Jörn Kalinowski, Andriy Luzhetskyy

**Affiliations:** ^1^Helmholtz-Institute for Pharmaceutical Research Saarland, Saarbrücken, Germany; ^2^Department for Pharmaceutical Biotechnology, University of Saarland, Saarbrücken, Germany; ^3^Center for Biotechnology (CeBiTec), Bielefeld University, Bielefeld, Germany

**Keywords:** *Streptomyces*, secondary metabolites, NRPS-trans-AT-polyketide synthase, stereochemistry, bioactivity

## Abstract

The environment of Lake Baikal is a well-known source of microbial diversity. The strain *Streptomyces* sp. IB2014/011-12, isolated from samples collected at Lake Baikal, was found to exhibit potent activity against Gram-positive bacteria. Here, we report isolation and characterization of linear polyketide alpiniamide A **(1)** and its new derivatives B–D **(2–5)**. The structures of alpiniamides A–D were established and their relative configuration was determined by combination of partial Murata’s method and ROESY experiment. The absolute configuration of alpiniamide A was established through Mosher’s method. The gene cluster, responsible for the biosynthesis of alpiniamides (*alp*) has been identified by genome mining and gene deletion experiments. The successful expression of the cloned *alp* gene cluster in a heterologous host supports these findings. Analysis of the architecture of the *alp* gene cluster and the feeding of labeled precursors elucidated the alpiniamide biosynthetic pathway. The biosynthesis of alpiniamides is an example of a rather simple polyketide assembly line generating unusual chemical diversity through the combination of domain/module skipping and double bond migration events.

## Introduction

The consistent development of antibiotic resistance in life-threatening pathogens diminishes the availability of effective medications for the treatment of infectious diseases. Natural products originating from plants and microorganisms have inspired medicinal drug research for decades (Ventola, 2015). They are produced as secondary metabolites and represent a major source of drug leads and serve as templates for semisynthetic derivatives. A vast number of secondary metabolites from microorganism, particularly from actinobacteria have been described to date. *Actinobacteria* represent one of the most thoroughly examined group of bacteria in terms of natural product research, which is reflected in the continuing discovery of important antibiotics, e.g., teicoplanin, daptomycin, and fidaxomicin over the years (Procópio et al., 2012). Many natural products not only serve as antibiotics but also as antiviral, immunosuppressive and even as anticancer agents (Vaishnav and Demain, 2011). Hence, the discovery of new bioactive natural products is still indispensable for sustaining the rapid progress of medicinal research (Dias et al., 2012).

A large proportion of biologically active secondary metabolites are polyketide derivatives. These compounds are assembled by polyketide synthases (PKSs), enzymes that conduct a simple repetitive condensation of acyl units to form poly-ß-ketide chains followed by conversion of the chain into structurally diverse metabolites. Depending on the structure and the enzymatic pathway, PKSs can be divided into three major types. Type I PKSs have modular architectures consisting of several modules each harboring ketosynthase (KS) and acyl carrier protein (ACP) domains accompanied by an acyltransferase domain (AT), which is responsible for the selection of the extender unit, as well as a set of processing enzymes (KR – ketoreductase, DH – dehydratase, and ER – enolreductase) (Khosla, 2009). Recently, a new type of modular PKS was discovered that lacked the acyltransferase domains within the modular proteins (Helfrich and Piel, 2016). The acyltransferase activity is a standalone protein that is typically encoded within the respective gene cluster. PKSs of this type are known as trans-AT and they are believed to be the most abundant type of secondary metabolite assembly lines.

Bacterial strains of the genus *Streptomyces* inhabit different ecological niches. They are found not only in soil but also in fresh water systems, marine habitats, isolated eco-systems, and in symbiosis with insects and plants (Hasani et al., 2014). Lake Baikal is a unique eco-system, rich in endemic species of living organisms. We have recently isolated a variety of actinobacteria strains from Lake Baikal (Axenov-Gribanov et al., 2016). Several of these strains were found to be active against Gram-positive and Gram-negative bacteria. The strain *Streptomyces* sp. IB2014/011-12 showed the most promising results when tested against *Bacillus subtilis*. This finding motivated us to analyze the strain in its entirety to identify the metabolites responsible for the observed activity.

Here, we report the activity-guided screening of metabolites produced by *Streptomyces* sp. IB2014/011-12 that resulted in the isolation of alpiniamide A **(1)**, which was previously described as a product of the endophytic *Streptomyces* sp. YIM66017 (Golinska et al., 2015), and its novel derivatives. The genome of this strain has been sequenced and analyzed, which has enabled us to identify the gene cluster responsible for the biosynthesis of alpiniamides.

## Materials and Methods

### Bacterial Strains, Culture Conditions, and General Procedures

The isolation and phylogenetic characterization of *Streptomyces* sp. IB2014/011-12 were reported in (Axenov-Gribanov et al., 2016). *Streptomyces* strains were grown on solid nutrient medium MS (mannitol soy flour agar) and in liquid TSB medium (Kieser, 2000). For secondary metabolite production, NL19 (MS medium without agar) and SG (glucose, yeast, Bacto Soytone, and calcium carbonate) medium have been used. *Escherichia coli* XL1Blue (Agilent, United States) was used for routine cloning, and *E. coli* MW 6026 was used as a donor in the intergenic conjugation (Blodgett et al., 2007). *E. coli* strains were grown in Luria-Bertani (LB) broth. For MW 6026, diaminopimelic acid was added. When required, antibiotics were added to the cultures at the following concentrations: 50 μg ml^−1^ apramycin, 100 μg ml^−1^ spectinomycin, 100 μg ml^−1^ phosphomycin, and 100 μg ml^−1^ carbenicillin (Sigma, United States; Roth, Germany).

### Recombinant DNA Techniques

Chromosomal DNA from *Streptomyces* strains and plasmid DNA from *E. coli* were isolated using standard protocols (Makar et al., 1975; Kieser, 2000). Restriction enzymes and molecular biology reagents were used according to the recommendations of the supplier (Thermo Fisher Scientific, Germany; NEB, **United States**).

### Genome Sequencing, Assembly, and Annotation

For DNA isolation, *Streptomyces* sp. IB2014/011-12 was inoculated into TSB medium and grown at 28°C with shaking (200 rpm) for 3 days. High quality total cellular DNA was isolated using salting out procedure. The purity and concentration of the genomic DNA was determined using a Nanodrop 2000 spectrophotometer (Thermo Fisher Scientific).

For sequencing of the *Streptomyces* sp. IB2014/011-12 genome, an Illumina paired-end sequencing library (TruSeq sample preparation kit; Illumina, United States) was constructed according to the manufacturer’s protocol. The *Streptomyces* sp. IB2014/011-12 draft genome sequence was established on an Illumina HiSeq system in rapid run mode (2 × 250 nt) with a pair distance of about 500 bp. Upon sequencing and processing of the obtained data, a *de novo* assembly was performed using the GS De Novo Assembler (version 2.8.) (Roche Diagnostics, Mannheim, Germany) with default settings. Annotation of the genome was performed by means of prokka v1.11 and the GenDB 2.0 platform (Meyer et al., 2003; Seemann, 2014). For the identification of secondary metabolites clusters antiSMASH 3.0 was used (Weber et al., 2015). The assembled and annotated draft sequence of the *Streptomyces* sp. IB2014/011-12 genome was deposited in the GenBank database under accession number QEIK00000000.

### Generation of the Construct for Gene Cluster Inactivation

Two DNA fragments, C9-1 and C9-2, flanking the first 2000 bp of the gene *alpA1* have been amplified using the primer pairs C9-2REcV and C9-2FEcI and C9-1FEcV and C9-1RXba (**Supplementary Table S1**). The obtained 2 kb fragments were cloned into a pST Blue-I AccepTor^TM^ vector (Novagen, United States). The construct containing fragment C9-1 was digested with *Eco*RV (primer) and *Xba*I (vector MCS) and ligated with the C9-2 fragment, which was retrieved with the same restriction enzymes. The resulting plasmid was digested with *Eco*RV and ligated with the spectinomycin-resistance cassette. The final construct was transformed into pKG 1132 (Myronovskyi et al., 2011). This construct was transferred into *Streptomyces* sp. IB2014/011-12 via an intergeneric conjugation. The exconjugants were grown under non-selective conditions and screened for white spectinomycin-resistant colonies when grown on MS supplemented with X-gluc (X-Gluc DIRECT, United States). The deletion of part of the *alpA1* gene was confirmed by PCR using the 11-12DelCheckF and 11-12DelCheckR primer pair (**Supplementary Table S1**).

### Cloning of the *alp*-Gene Cluster and Red/ET Mediated Gene Deletion

The two 2.5 kb DNA fragments, Tar1 and Tar2, flanking a 46.7 kb region of the *Streptomyces* sp. IB2014/011-12 chromosome that includes the entire *alp*-gene cluster were amplified with the primer pairs 11-12C9TarF1Not and 11-12C9TarR1Nhe and 11-12C9TarF2NheI and 11-12C9TarR2HindIII, respectively (**Supplementary Table S1**), using Phusion DNA polymerase and cloned into the pJET1.2 vector (Thermo Fisher Scientific, **United States**). Fragments were assembled by digesting the Tar1-containing plasmid with *Nhe*I (primer) and *Hind*III (vector MCS) and ligating with the *Nhe*I/*Hind*III-retrieved Tar2 fragment. The resulting construct was sub-cloned into a *Nhe*I/*Hind*III-digested pCLY10 vector (Bilyk et al., 2016). The final construct was linearized with *Nhe*I and mixed with *Streptomyces* sp. IB2014/011-12 chromosomal DNA in a 1:5 ratio. The mixture was transformed into *S. cerevisiae* BY4742 (Winston et al., 1995) with the standard LiAc protocol (Gietz and Schiestl, 2007). Transformants were selected on YNB medium supplemented with yeast synthetic drop-out medium supplements without leucine (Sigma-Aldrich, United States). Colonies were plated in patches of 100, washed, and analyzed by PCR for the presence of clones harboring the desired construct using the primers 11-12C9CheckHind (**Supplementary Table S1**) annealed to the cloned region outside of the homology fragment and pCLYCheckHind (**Supplementary Table S1**) annealed to the vector. The positive clone was further pooled out, the total DNA was purified using a standard protocol (Green and Sambrook, 2012) and transformed into *E. coli* XL1Blue to give 011-12p1-49 clone carrying the desired region of the *Streptomyces* sp. IB2014/011-12 chromosome. 011-12p1-49 plasmid DNA was purified and sequenced by MinION (Oxford Nanopore, United Kingdom). 011-12p1-49 was introduced into *S. lividans* TK24 and *S. albus* Del14 (unpublished data, Dr. M. Myronovskyi, personal communication) by intergeneric conjugation. The resulting strains were grown, and the production of alpiniamides was analyzed as described below. *alpD*, *alpR*, and *alpE* genes were inactivated by replacement with a hygromycin resistance cassette from plasmids patt-shyg (Myronovskyi et al., 2011) within the 011-12p1-49 construct. λ-Red recombineering was performed as described (Gust et al., 2004). Primers used to amplify the cassette and to verify the deletions are listed in **Supplementary Table S1**. The resulting constructs were introduced into the host *S. albus* Del 14 via conjugation and clones were selected with hygromycin. The mutants were cultivated in NL19 and production was analyzed using LC-MS as described below.

### Production, Extraction and LC-MS Analysis of Alpiniamides

The *Streptomyces* sp. IB2014/011-12 was grown in 10 ml of TSB for 2 days at 28°C on a rotary shaker. The main culture (100 ml of NL19 in 500 mL flasks with glass beads) was inoculated with 1 ml of the pre-culture and cultivated at 28°C and 180 rpm for 7 days. The metabolites in the cultural liquid were extracted with ethyl acetate and from the biomass with a mixture of acetone and methanol (1:1). The solvents were evaporated, and the residue was dissolved in 300 μl of methanol.

The LC-MS data were collected on a Dionex Ultimate 3000 RSLC system using a BEH C18, 100 × 2.1 mm, 1.7 μm d_p_ column (Waters, Germany). Separation of a 1 μl sample was achieved by a linear gradient of solvent B (acetonitrile with 0.1% of formic acid) against solvent A (water with 0.1% of formic acid) at a flow rate of 600 μl/min and 45°C. The gradient started with a 0.5 min isocratic step of 5% B then increased to 95% B over 18 min and ended with a 2 min step of 95% B before re-equilibration under the initial conditions. UV spectra were acquired by a DAD in the range of 200 to 600 nm. The mass spectrometry data were collected on an amazon SL speed mass spectrometer (Bruker Daltonics, Germany) using an Apollo II ESI source. Mass spectra were acquired in centroid mode ranging from 200 to 2000 *m/z* at a scan rate of 2 Hz. The HRMS data were collected on a Dionex Ultimate 3000 RSLC system using a BEH C18, 100 × 2.1 mm, 1.7 μm d_p_ column (Waters, Germany). Separation of a 1 μl sample was achieved by a linear gradient of solvent B (acetonitrile with 0.1% of formic acid) against solvent A (water with 0.1% of formic acid) at a flow rate of 480 μl/min and 45°C. The gradient started with a 0.5 min isocratic step of 5% B then increased to 95% B over 20 min and ended with a 2 min step of 95% B before re-equilibration under the initial conditions. UV spectra were acquired by a DAD in the range of 200 to 600 nm. High-resolution mass spectrometric data were collected on an LTQ Orbitrap mass spectrometer (Thermo Fischer Scientific, United States).

Data were collected and analyzed with the Bruker Compass Data Analysis software, version 4.2 (Bruker, Billerica, MA, United States) and the Thermo Xcalibur software, version 3.0. The screening for known compounds was performed using the Dictionary of Natural Products Database, version 10.0 (CRC Press, Boca Raton, FL, United States), using the following parameters: accurate molecular mass, absorption spectra and biological source (Running, 1993). Compounds were considered to be similar when the difference in accurate mass was less than 2 ppm and the absorption spectra were identical.

### Feeding Experiments

Thirty milliliter of liquid NL19 medium was inoculated with 300 μl of a 22 h old seed culture of the strain *Streptomyces* sp. IB2014/011-12. After 10 h of growth on a rotary shaker at 28°C, the culture was supplemented with 200 μl of Glycine-2-^13^C or 200 μl methionine(-methyl-^13^C) respectively, solved in water (5.2 mg/ml). This procedure was repeated four times every 10–12 h. The final concentration of compounds in the flask was 2.31 mM of Glycine-2-^13^C and 1.16 mM of methionine(-methyl-^13^C). After 7 days of cultivation, the culture was extracted separately, biomass with acetone/methanol (1:1) and the supernatant with ethyl acetate. The solvent was evaporated and the obtained residues solved in MeOH. The extracts were subjected to LC-MS analysis.

### Isolation and Structure Elucidation of Alpiniamides

For the isolation of the metabolites, the strain was cultivated as described above in 8 L of NL19. Metabolites were extracted from cultural liquid with equal volume of ethyl acetate, solvent was evaporated and resulting 1.56 g of crude extract was dissolved in 8.5 ml of methanol. The crude extract was purified through size-exclusion chromatography using Sephadex^®^ LH 20 (Sigma-Aldrich) and MeOH as eluent (1 m long column with 700 ml volume of Sephadex). Fractions were collected every 15 min with a speed of 1-2 drops per second (approximately 30 ml per hour). The obtained fractions with antibacterial activity were further purified by preparative and subsequent semipreparative high-performance-liquid-chromatography (HPLC) using the following equipment: Dionex Ultimate 3000 from ThermoScientific (preparative) and Agilent 1260 Series and 1100 Series from Agilent Technologies (semipreparative). For preparative HPLC, a Nucleodur C18 HTEC column (150 × 21 mm, 5 μm) was used with a multistep gradient from 15–20% B (B: acetonitrile with 0.1% formic acid; A: water with 0.1% formic acid) over 2 min and 20–60% B over 20 min at a flow rate of 20 ml/min and 45°C. Semipreparative HPLC was performed using a Synergi Phusion RP-Column (250 × 10 mm, 4.6 μm; Phenomenex) with a gradient elution from 5 to 95% B (B: methanol with 0.1% of formic acid; A: water with 0.1% formic acid) over 20 min at a flow rate of 4.5 ml/min and 45°C. UV spectra were recorded with a DAD detector at 200–600 nm.

NMR spectra were acquired in deuterated methanol (CD_3_OD) and deuterated chloroform (CDCl_3_) at 298 K on a Bruker Avance III 700 or 500 MHz spectrometer, both equipped with a 5 mm TXI cryoprobe. NMR shifts were relative to the residual solvent signal CH_3_OD at δ 3.30 and CDCl_3_ δ 7.24 for ^1^H, or to the solvent itself at δ 49.00 (CD_3_OD) and 77.00 (CDCl_3_) for ^13^C measurements. NMR data were analyzed using Topspin, version 3.5 pl7 (Bruker, United States).

### Preparation of the S- and R-MTPA-Ester of Alpiniamide A (6S and 6R), Alpiniamide B_1_ (7S and 7R), and Alpiniamide B_2_ (8S and 8R) by the Modified Mosher’s Method

To a solution of alpiniamide A (**1**, 0.5 mg, 1.45 μmol) and dry pyridine (20 μl) in dry deuterated chloroform (100 μl) at room temperature, α-methoxy-α-(trifluoromethyl)phenylacetyl chloride (R-MTPA-Cl) (20 μl, 0.10 mmol) was added. The reaction was mixed at room temperature for 4 h. Another 200 μl of CDCl_3_ was added, and the sample now containing the S-MTPA-ester of alpiniamide A (**6S**) was directly subjected to NMR measurements (**Supplementary Figure S1**). The R-MTPA-ester of alpiniamide A (**6R**) was prepared in the same way using S-MTPA-Cl instead of its R-isomer. The same procedure was carried out with only a fifth of all ingredients to get **7S** and **7R** from alpiniamide B_1_ **(2)** and **8S** and **8R** from alpiniamide B_2_ **(3)**, respectively (**Supplementary Figure S1**).

### Bioactivity Assay

The antimicrobial activities of isolated compounds were measured by a disk diffusion assay. A 30 μl aliquot of each compound was loaded on a paper disk with a diameter of 6 mm, allowed to dry, and set on an LB agar plate coated with *B. subtilis* from liquid overnight culture. The plates were incubated at 37°C for 12 h. The zones of inhibition were measured manually. The minimal inhibitory concentrations were estimated by standard serial dilutions protocol in 200 μL in 96 well plates. Briefly, serial dilutions (1:1) of alpiniamides were prepared using DMSO as a solvent prior to aliquoting 10 μL of each solution into 96 well plate. Kanamycin was used as a positive control (full inhibition of growth), and DMSO was used for the negative control (full growth). 190 μL of bacterial test cultures in appropriate media (1:500 dilution of overnight culture) were added to each well, and the plates were shaken at 30°C for 16-20 h. Then, 5 μL of thiazolyl blue tetrazolium bromide (10 mg mL^−1^) solution was added to each well, and the plates were incubated at 30°C for an additional hour. MICs were determined as the concentration of antibiotic in the well where the additive did not change the color of the solution from yellow to dark blue.

## Results

### Genome Analysis

S*treptomyces* sp. IB2014/011-12 has been isolated from the net-spinning caddisfly *Trichoptera* sp. (larvae) (Axenov-Gribanov et al., 2016). Phylogenetic analysis based on the 16 sRNA showed that the strain IB2014/011-12 belongs to the genus *Streptomyces*. The strain grows as a typical actinobacteria forming colonies with aerial mycelia and gray spores (**Supplementary Figure S2**). The strain accumulates a brownish pigment during growth on a solid medium. When grown in liquid media (NL19) the strain demonstrated activity against Gram-positive bacteria. The genome of *Streptomyces* sp. IB2014/011-12 has been sequenced and assembled into 71 contigs (**Table 1**). The largest contig is 956.9 kbp. The overall size amounts to 8099.23 kbp, which is in the normal range for streptomyces. The genome consists of a single chromosome and has no extra chromosomal DNA based on the sequence coverage. The genome has a G + C content of 71.5%. The chromosome is predicted to contain 7.282 coding sequences, 5 rRNA clusters, 80 tRNA genes and one tmRNA gene (Palecková et al., 2009). The analysis of the genome using antiSMASH software revealed that 29 gene clusters are involved in the biosynthesis of diverse secondary metabolites including terpenes, lanthipeptides, non-ribosomal peptides, and polyketides (**Supplementary Table S2**).

**Table 1:** Features of *Streptomyces* sp. IB2014/011-12 genome.

Genome size	8099.23 bp
Contigs	71
GC content	71.5%
CDS	7.282
tRNA	80
mRNA	1
rRNA	5
Cluster	29

Based on the gene cluster analysis with antiSMASH the production of several secondary metabolites by *Streptomyces* sp. IB2014/011-12 can be predicted (**Supplementary Table S2**). Like most of actinobacteria strains, the genome contains gene clusters for desferrioxamine, ectoine, and melanin production. Desferrioxamine B, a high-affinity iron chelator (siderophore), is important for scavenging ferric iron as a nutrient for the cells (Barona-Gómez et al., 2004). Ectoine, another common metabolite, is an osmoprotectant (Zhu et al., 2014). The function of melanin, a black pigment, that is produced by many types of bacteria, is still under discussion (Woo et al., 2010). The compound is most likely involved in protection against chemical and biological stresses, such as exposure to heavy metals, oxidizing agents and UV radiation (Allam, 2012), and it might be the reason why the MS medium is colored by that strain (**Supplementary Figure S2**). In addition, the gene cluster that produces the class III lantipeptide AmfS is present in the genome of *Streptomyces* sp. IB2014/011-12. The predicted amino acid sequence of the precursor peptide from the gene cluster coincides with the sequence of AmfS isolated from *Streptomyces griseus* (Ueda et al., 2002). AmfS-like compounds are known to be positive regulators of the formation of aerial-mycelium in streptomyces. Another secondary metabolite that could be predicted from the *Streptomyces* sp. IB2014/011-12 genome analysis is roseoflavin. This compound belongs to the riboflavin antibiotic family, which targets riboswitches that affect bacterial growth (Mansjö and Johansson, 2011; Schwarz et al., 2016). Furthermore, the gene cluster no. 3 is probably coding for the class II lasso peptide SRO15-2005 since it shows 100% sequence homology to the corresponding gene cluster of *S. roseosporus* NRRL 15998 (Kersten et al., 2013).

### Dereplication of Secondary Metabolites Produced by *Streptomyces* sp. IB2014/011-12

**FIGURE 1:**
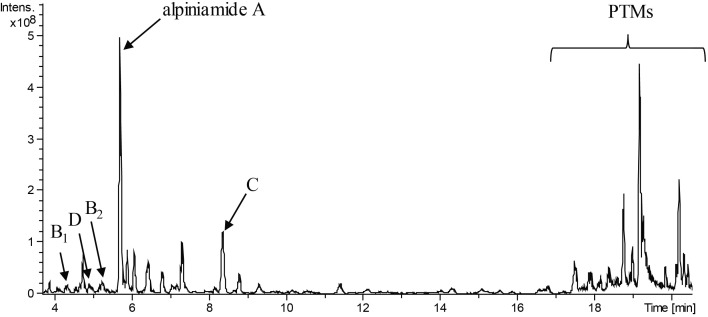
LC-MS chromatogram (minute 4 to 20 from an overall 20 min run) of extract of *Streptomyces* sp. IB2014/011-12 cultivated in NL19 medium. The peaks for different alpiniamides are marked with letters. Peaks for polycyclic tetramate macrolactams are highlighted with the bracket.

Dereplication is a quick and simple method of analyzing metabolites based on LC-MS data. It allows known compounds to be distinguished from potentially new metabolites to avoid their purification and analysis. With the help of UV/Vis spectra, high-resolution mass spectrometric data, biological source and other criteria, one can compare entries in data banks such as the “Dictionary of Natural Products (DNP)” in order to identify metabolites in the extract of interest (Whittle et al., 2003). At first, to estimate the potential novelty of the compounds produced by *Streptomyces* sp. IB2014/011-12, we analyzed the LC-MS data of the extracts of the strain grown in NL 19 media. The obtained exact masses from MS data were further compared to the DNP. This led to the identification of some known compounds and prediction of putative new metabolites produced by this strain (**Figure 1**). The major class of compounds produced by this strain is polycyclic tetramate macrolactams (PTM) (**Supplementary Figures S3**, **S4**) (Xu et al., 2015). We identified alteramide A **(9)** (Shigemori et al., 1992) and several isomers that were not distinguishable since they all have *m/z* 511.28207 [*M*+H]^+^ (510.27427 [*M*], calculated 510.2730) ions and UV curves typical for PTMs but different retention times. Under the used conditions, the isomers elute at RTs of 17.7, 18.6 and 19.0 min. Alteramide B **(10)** and several isomers with *m/z* 495.28549 [*M*+H]^+^ (494.27769 [*M*], calculated 494.2781) were present in the extract as well. They eluted at RT 18.3, 18.9, 19.3, and 19.5 min. In addition to these, the strain also produces clifednamide A **(11)** (*m/z* 509.26395 [*M*+H]^+^, 508.25615 [*M*] calculated 508.2573, RT at 18.4) and clifednamide B **(12)** (*m/z* = 493.27017 [*M*+H]^+^, 492.26237 [*M*] calculated 492.2624, RT at 18.7) (Cao et al., 2010) and possible isomers of these compounds that eluted at different retention times (RT at 18.8 and 18.9 min). Furthermore, the compound eluting at RT 18.2 min with *m/z* 513.29565 [*M*+H]^+^ (512.28785 [*M*], calculated 512.2886) corresponds to dihydromaltophylin **(13)** (heat-stable antifungal factor, HSAF) (Li et al., 2008).

Alteramide A and B, isolated from marine bacterium *Alteromonas species*, are macrocyclic lactams that contain a dienone and a dienoyl tetramic acid moiety (Shigemori et al., 1992; Ding et al., 2016). They exhibit activity against leukemia cells but are not active against bacteria. Dihydromaltophilin is similar to alteramide in that it also has three five-membered rings. The compound was isolated from *S. maltophilia* R3089 and exhibits activity against a broad spectrum of fungi but is not active against Gram-positive or Gram-negative bacteria (Jakobi et al., 1996). The clifednamides, another group of PTMs, were first isolated from *Streptomyces* sp. JV178. The biological activity of these compounds has not yet been published. In the genome of *Streptomyces* sp. IB2014/011-12, a hybrid type I PKS-NRPS cluster no. 13 has been identified, and it is similar to the known gene clusters for PTMs biosynthesis (**Supplementary Figure S5**). The biosynthetic gene cluster for alteramide, clifednamide and dihydromaltophilin differ slightly in their structures. Cluster no. 13, with the core gene coding for iterative hybrid NRPS-type I PKS, provide all necessary enzymatic activities for PTM production (CDCs: 14650–14675, **Supplementary Figure S5**). The gene cluster encodes for a putative hydroxylase (14675), an iterative type 1 PKS-NRPS (14670), two oxidoreductases (14665–14660), alcohol dehydrogenase (14655), and cytochrome P450 hydroxylase (14650) in the same arrangement as in the biosynthetic gene cluster for HSAF and frontalamides (Lou et al., 2011).

### Isolation and Structure Elucidation of Alpiniamide A–D (1–5)

**FIGURE 2:**
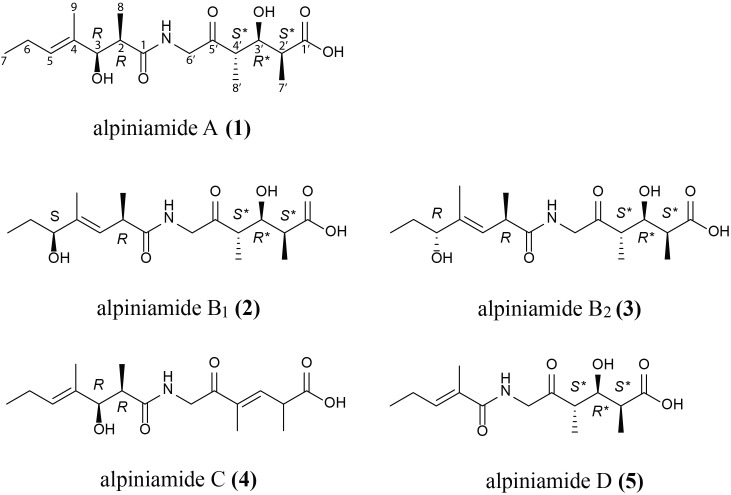
Revised structure of alpiniamide A **(1)** and structures of new alpiniamides B-D **(2–5)** from *Streptomyces* sp. IB2014/11-12.

*Streptomyces* sp. IB2014/011-12 produced additional metabolites (**Figure 1**) that we could not dereplicate against known products. For this reason, we assumed that the activity was caused by potentially new metabolite(s). We grew the strain in 8 L of NL19 and extracted the produced metabolites. Subsequently, a bioactivity-guided purification was carried out by tracking down the bioactive compounds using the disk diffusion assay at each step of purification. The crude extract was first fractionated using size-exclusion chromatography, and the obtained fractions were tested against *B. subtilis*. The active fractions were combined and further purified with preparative HPLC and then with semipreparative HPLC. The fractions collected after each separation step were tested for antibacterial activity. This resulted in the isolation of five compounds. The structure elucidation showed that they are alpiniamides (**Figure 2**).

**Alpiniamide A (1)** was obtained as a slightly red solid (4 mg), with a molecular formula of C_17_H_29_NO_6_ as determined by high-resolution electrospray ionization mass spectrometry (HRESIMS) at *m/z* 344.20596 [*M*+H]^+^, indicating 4 degrees of unsaturation. Its NMR spectra, acquired in CD_3_OD (**Supplementary Table S3**) revealed 17 carbons, five methyls (one adjacent to a double bond), two methylenes, six methines (two secondary alcohols and one as part of a double bond), and four quarternary carbons (three carbonyls and one as part of a trisubstituted double bond). The chemical shifts of the methylene group at δ_H_ 4.13 and 4.25 and δ_C_ 49.0 indicated the presence of an amide nitrogen atom nearby. These data together with the results of 2D ^1^H-^1^H-COSY, HSQCED, HMBC, and ROESY led to the structure of alpiniamide, which was previously isolated from the *Streptomyces* sp. YIM660107 (Zhou et al., 2013), with NMR data recorded in CDCl_3_. In order to compare the literature data with our data, we reran the 1D NMR spectra of **1** in CDCl_3_ (**Supplementary Table S3** and **Supplementary Figures S6**, **S7**). The obtained data were close to those from the reported structure. But the remarks to the relative stereochemistry of alpiniamide were confusing. C-2/C-3 was found to be *threo*, but the structure of alpiniamide showed the *erythro*-form. C-2′/C-3′/C-4′ was announced as *threo* as well, which is ambiguous and most likely a wrong phrasing for a system with three chiral centers. However, the structure showed C-2′/C-3′ and C-3′/C-4′ both in *erythro*-configuration. Therefore, we were motivated to investigate the stereochemistry of **1** in more detail.

Due to the large vicinal coupling constant of 9.5 Hz for *J*_H2H3_, the configuration for C-2/C-3 in part A (C-1 to C-9) was found to be *threo*. We are aware, that a single coupling constant cannot usually distinguish which of two diastereomers might be present since there are three possible staggered conformations for each diastereomer, two of which will typically have very similar predicted coupling constants for a pair of vicinal protons. But assignments become possible when one can make some reliable predictions on which conformation predominates. Such a situation is given for part A of **1**, where the hydroxyl at C-3 can form an intramolecular hydrogen bond with carbonyl C-1 when measured in the non-polar solvent CDCl_3_. Then, the ^3^*J*_HH_ coupling constant of two vicinal protons are considerably large when they are in the *threo* configuration. Small values for ^3^*J*_HH_ indicate the *erythro* form (Stiles et al., 1964; House et al., 1973). A similar situation predominates in part B between the hydroxyl at C-3′ and the carbonyls C-1′ and C-5′. Careful analysis of ^3^*J*_HH_ coupling constants led to 7.0 Hz for *J*_H2′H3′_ and 4.5 Hz for *J*_H3′H4′_. The small coupling constant for H-3′/H-4′ give rise to an *erythro* configuration, whereas the moderate high *J* value for H-2′/H-3′ is a hint for the *threo*-form (**Supplementary Table S3**) (Xu et al., 2017). Nevertheless, a final proof of the relative stereochemistry of **1** requires a complete set of ^3^*J*_HH_ and ^2,3^*J*_HC_ coupling constants as shown in Murata’s method (Matsumori et al., 1999; Bifulco et al., 2007). Due to the limited amount of **1** and its insufficient stability during storing, we were not able to perform it.

In order to determine the absolute configuration as well, we applied Mosher’s method (Dale and Mosher, 1973; Hoye et al., 2007). Portions of **1** were separately treated with (R)-MTPA-Cl and (S)-MTPA-Cl to yield the Mosher ester **6S** and **6R**, respectively (**Supplementary Figure S1**). The differences in the proton chemical shifts of **6S** and **6R** (Δδ_(S-R)_) should give positive or negative values from which the configuration can be established. For part A of the molecule we obtained negative values for H-2 and H-8 and positive values for H-5, H-6, H-7, and H-9 (**Supplementary Table S4** and **Supplementary Figure S8**). On the basis of these results, the absolute configuration can be determined as *R* at C-3. From the relative configuration, the neighboring positions can also be assigned, resulting in *R*-configuration at C-2. The results for part B are less clear. We obtained negative values for H-2,’ and positive values for H-4,’ H-7,’ and H-8’. For an appropriate interpretation H-2’ and H-7’ should have one sign and H-4’ and H-8’ the opposite sign. However, H-7’ does not coincide with this rule and makes therefore a distinct statement about the absolute configuration for H-3′ impossible. Such issues with the assignment of the absolute configuration with MTPA are known for linear secondary alcohols (Seco J.M. et al., 2004). The reason for inconsistent sign distribution and small Δδ_(S-R)_ values often lies in the presence of different conformers of the MTPA ester. Theoretical calculations revealed that a rotation about the C_α_ – CO and C_α_ – Ph bond generates three conformers (Latypov et al., 1996). All three conformers are present in similar populations and each conformer has a different shielding/deshielding effect, and therefore it influences the final spectrum in different ways. This information together with the inconsistent results from Mosher’s method for the identical parts of the close related compounds **2** and **3** (see below) hinders the assignment of the absolute configuration for C-3′. Alternative reagents, e.g., methoxyphenylacetic acid (MPA) or α-(9-anthryl)-α-methoxy-acetic acid (9-AMA) should give more precise values (Seco J.M. et al., 2004). Due to the little amount of **1** we had to refrain from further efforts to determine the absolute configuration for this part of the molecule.

**FIGURE 3:**
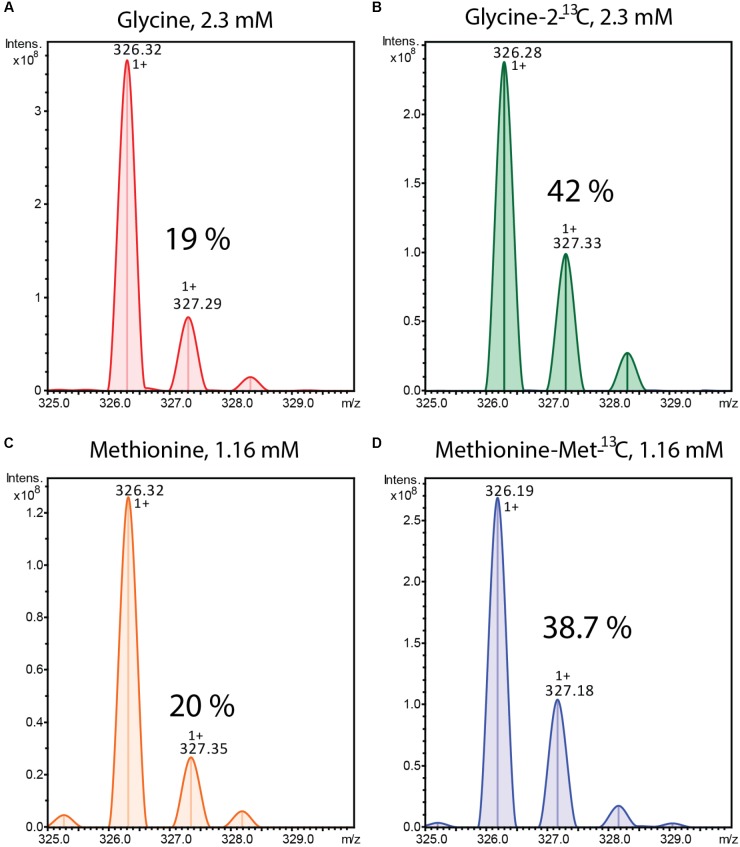
Mass spectra of alpiniamide A (*m/z* 326.32 [*M*-H_2_O+H]^+^) extracted from *Streptomyces* sp. IB2014/011-12 culture cultivated in the presence of unlabelled **(A)** and ^13^C labeled **(B)** glycine and unlabelled **(C)** and ^13^C labeled **(D)** methionine. The % of isotope containing ions is indicated.

Therefore, the structure of **1** was established as shown in **Figure 3**. We named it alpiniamide A, due to the close relationship to alpiniamide from a *Streptomyces* species mentioned above. It is not excluded that alpiniamide and alpiniamide A have identical structures. But due to the confusion concerning the relative configuration of alpiniamide in the literature and the lack of coupling constants especially for H-2′-H-4′, we were not able to determine it.

**Alpiniamide B_1_ and B_2_ (2 and 3)**, were both isolated as red solids (0.5 mg), with a molecular formula of C_17_H_29_NO_6_ as determined by their HRESIMS data (*m/z* 344.20596 [*M*+H]^+^). The ^1^H NMR data (**Supplementary Table S3** and **Supplementary Figures S9**, **S10**) of both epimers were very close to each other and to those found for **1**. But in contrast to it, the double bond in part A of **2** and **3** was shifted from C-4 to C-3 and the secondary alcohol from C-3 to C-5. The only difference between alpiniamide B_1_ and B_2_ was found in the stereochemistry of the secondary alcohol at C-5. When applying Mosher’s method to both isomers, the stereocenter at C-5 was assigned as *S* in alpiniamide B_1_ and *R* in alpiniamide B_2_ (**Figure 2**, **Supplementary Table S4**, and **Supplementary Figure S8**). Due to the high conformity in the chemical shifts of H-2 and its neighboring atoms the configuration at C-2 is the same in both molecules and most likely *R* as it is given for alpiniamide A. The *E*-geometry of the double bond C-3/C-4 was confirmed by ROESY measurements with key correlation between double bond proton H-3 and the H-5 of the secondary alcohol. Part A is identical in all three alpiniamides. Even the coupling constants for H-2′, H-3,′ and H-4′ of the three compounds were identical indicating the same stereochemistry for this substructure in all three molecules. As for alpiniamide A, the determination of the absolute stereochemistry via Mosher’s method failed for **2** and **3** due to the anomaly of the detected Δδ(_S-R_) values (**Supplementary Figure S8**).

**Alpiniamide C (4)** was obtained as a white solid (0.8 mg) with a molecular formula of C_17_H_27_NO_5_ as determined from its HRESIMS at *m/z* 326.19589 [*M*+H]^+^. The mass difference of 18 units compared to **1–3** indicated the loss of a water molecule. The ^1^H and ^13^C resonances (**Supplementary Table S3** and **Supplementary Figure S11**) lacked one methine and a secondary alcohol function. Instead, resonances for an additional trisubstituted double bond (δ_H_ 6.87; δ_C_ 134.8 and 147.0) appeared in the spectra. The chemical shifts of part A were close to those of **1**. Therefore, the additional double bond must be located in part B. Its position between C-3′ and C-4′ was established by HHCOSY correlations starting from methyl H-7′ (δ_H_ 1.29 d, 7.0 Hz) via H-2′ (δ_H_ 3.40 m) to H-3′ (δ_H_ 6.87 d, 9.5 Hz).

**Alpiniamide D (5)** is the derivative with the lowest molecular mass. It is a colorless solid (0.4 mg) with a molecular formula of C_14_H_23_NO_5_ as determined from its HRESIMS at *m/z* 286.16458 [*M*+H]^+^. Due to its NMR data, part B of **5** is identical to that of **1**. However, signals for methines C-2 and C-3 and methyl C-8 in part A were missing in the spectra of **5** (**Supplementary Table S3** and **Supplementary Figure S12**) and gave therefore a hint of their deletion in part A. Vicinal HMBC correlations from carbonyl C-1 (172.2) to the double bond proton at δ_H_ 6.39 and the adjacent methyl protons at δ_H_ 1.84 supported this observation and led to structure **5** for alpiniamide D.

Alpiniamide A was previously isolated from the *Streptomyces* sp. YIM 66017. The crude extract of that strain was tested against the *Bacillus anthracis* and fungi *Fusarium solani* (Zhou et al., 2013). In both cases inhibition zones were observed. Hence, we have tested the known alpiniamide A and its derivatives C and D on a panel of bacterial and yeast test cultures. The minimal inhibitory concentration (MIC) was determined against the Gram positive bacteria *Staphylococcus carnosus* DSMZ 20501, *Kocuria rhizophila* DSMZ 348, *Enterococcus mundtii* DSMZ 4840, *Micrococcus luteus* DSMZ 1790, *Mycobacterium smegmatis* DSMZ 43286, and *B. subtilis* DSMZ 10, against the Gram negative bacteria *Erwinia persicina* DSMZ 19328 and *Pseudomonas putida* KT2440 and against yeast *Candida glabrata* DSMZ 11226. In all cases we did not observe growth inhibitory activity in the concentration range up to 100 μg/ml. Also, pure compounds were found to be not active against *B. subtilis* in disk diffusion test. Since the mixture of all alpiniamides in the last stage of the purification was clearly active against *B. subtilis* we assume that the inhibitory concentration of alpiniamides lies above 100 μg/ml or the observed antibacterial activity is caused by synergistic effect with some minor compound(s).

### Feeding Experiments

From the structures of the isolated compounds we can predict that they are synthesized by the combined action of NRPS and PKS enzymes. The left and the right sides of the amide bond are typical products for a type I PK, whereas the peptide bond in the middle most likely arises from the amino acid glycine introduced by an NRPS enzyme. The methyl groups of the alpiniamides could originate from the direct use of methylmalonate during polyketide assembly or from a secondary methylation event with S-adenosyl methionine (SAM) as a donor. We grew two cultures of *Streptomyces* sp. IB2014/011-12 fed with ^13^C-labeled glycine and with ^13^C-labeled methionine (methyl-^13^C), respectively. The glycine was fed for the first time at 10 h post-inoculation. Methionine was fed for the first time at 36 h after inoculation. For both compounds, the feeding was repeated four more times every 10 h resulting in final concentration 2.31 mM of Glycine-2-^13^C and 1.16 mM of methionine(-methyl-^13^C). As a control, the culture supplemented with the corresponding unlabeled compound was used. The metabolites were extracted and analyzed by LC-MS. As result, the +1 isotopic peaks of all alpiniamides in the ^13^C-labeled glycine culture increased in intensity (42%) indicating the successful incorporation of the compound (**Figures 3A,B**). In contrast, the +1 isotopic peaks of alpiniamides in the culture supplemented with unlabeled glycine were observed at their normal intensity (19%). In the case of methionine, the results demonstrated the successful incorporation as well. The +1 isotopic peak of alpiniamides in the ^13^C-labeled methionine culture reached an intensity of 38.7% (**Figures 3C,D**). The +1 isotopic peaks of alpiniamide in the unlabeled culture appeared at their normal intensity of 20%. These results suggest that glycine is incorporated intact into alpiniamides and is the source of the amino group. On the other hand, we confirmed that at least some methyl groups originate from the SAM-dependent C-methyltransferase activity rather than methylmalonate.

### Alpiniamide Gene Cluster Inactivation and Heterologous Expression

**FIGURE 4:**
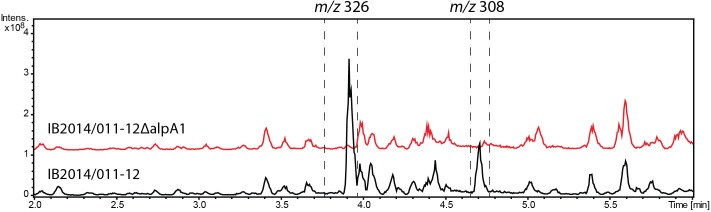
Production of alpiniamides A-B (*m/z* 326 [*M*-H_2_O+H]^+^) and alpiniamide C (*m/z* 308 [*M*-H_2_O+H]^+^) by *Streptomyces* sp. IB2014/011-12 and its mutant strain IB2014/011-12ΔalpA1 lacking *alpA1* gene. LC-MS chromatogram RT 2–6 min out of an overall 9 min gradient run is shown. Black line – extract of wild type *Streptomyces* sp. IB2014/011-12 and red line – extract of mutant strain lacking part of *alpA1* gene and its promoter. Strains were cultivated for 7 days in NL19 medium. The peaks for alpiniamides are highlighted. Alpiniamide D elutes under these conditions together with main alpiniamides A and B.

The labeled substrate feeding tests suggest that the alpiniamides probably arise from the action of a hybrid PKS/NRPS assembly line that facilitates the incorporation of glycine. The only gene cluster in the genome of *Streptomyces* sp. IB2014/011-12 that corresponds to the hypothesized biosynthetic scheme is no. 9 (**Figure 6A** and **Table 2**). This cluster encodes a hybrid NRPS-trans-AT-PKS that consists of four ketosynthase and one NRPS modules and a single NRPS module with an adenylation domain predicted to be specific for glycine as substrate. Two PK modules contain SAM-dependent C-methyltansferase domains. Overall, the architecture of the PKS-NRPS enzymes of cluster no. 9 suggests that it might be involved in the biosynthesis of alpiniamides. To verify this assumption, we replaced 2 kb of gene 12750 encoding the first PKS megaenzyme and its promoter with the spectinomycin-resistance cassette. The deletion was achieved through targeted gene disruption via double homologous recombination. The utilization of the pKG1132 suicide vector simplified the selection of colonies with double crossover by blue-white phenotypes due to the presence of the *gusA* gene in the vector backbone (Myronovskyi et al., 2011). The mutant strain and the wild type were cultivated in NL19 production medium, and the produced metabolites were analyzed by LC-MS. Compared to the wild-type strain, the mutant IB2014/011-12ΔalpA1 completely lacks the ability to produce alpiniamides (**Figure 4**). This proves that gene cluster no. 9, designated *alp*-cluster (**Table 2**), is indeed responsible for the biosynthesis of alpiniamides.

**Table 2:** Description of alpiniamide biosynthesis gene cluster and deduced function of *alp*-genes.

ID	Name	Predicted function	Closest characterized homologs (% of identical amino acids)	GenBank accession number
12715	*alpI*	fatty acyl-CoA reductase	putative short chain dehydrogenase, hitachimycin, 46% (*Streptomyces scabrisporus*)	LC008143
12720	*alpH*	TetR family transcriptional regulator	^∗^TetR/AcrR family; *Streptomyces* sp. IB2014 011-1 (100%)	-
12725	*alpW*	transporter, ATP binding protein	UvrA-like protein, quinomycin, 76% (*Streptomyces griseovariabilis*)	JN852959
12730	*alpG*	glyoxalase protein	putative lyase, pentalenolactone, 82% (*Streptomyces avermitilis*)	BA000030
12735	*alpF*	transcriptional activator/DNA repair enzyme	putative AraC-family transcriptional regulator, pentalenolactone, 86% (*Streptomyces avermitilis*)	BA000030
12740	*alpT*	malonyl-CoA ACP transacylase	acyltransferase/oxidoreductase, chivosazole, 50% (*Sorangium cellulosum*)	DQ065771
12745	*alpE*	3-oxoacyl-ACP synthase 3	3-oxoacyl-ACP synthase, cosmomycin D, 48% (*Streptomyces olindensis*)	JJOHO1000002
12750	*alpA3*	PKS: KS, AT, ACP; KS, DH, KR, ACP	polyketide synthase, kijamimicin 48% (*Actinomadura kijaniata*)	EU301739
12755	*alpA2*	PKS: KS, ACP, cMT, TE	polyketide synthase, bacillaene, 38% (Bacillus amyloliquefaciens)	AJ634060
12760	*alpA1*	PKS/NRPS: C, A, PCP; KS, ACP, cMT; KS, DH, tDH, KR, ACP	OnnI, Onnamide, 44% (*Candidatus Entotheonella* sp.*)*	AY688304
12765	*alpD*	acyl-CoA dehydrogenase	^∗^acyl-CoA dehydrogenase, dutomycin, 88% (*Streptomyces minoensis*)	KP710956
12770	*alpR*	ribosomal RNA large subunit methyltransferase G	methyltransferase containing protein; *Streptomyces* sp. IB2014 011-1 (100%)	-

*^∗^shown are closest homologs.*

**FIGURE 5:**
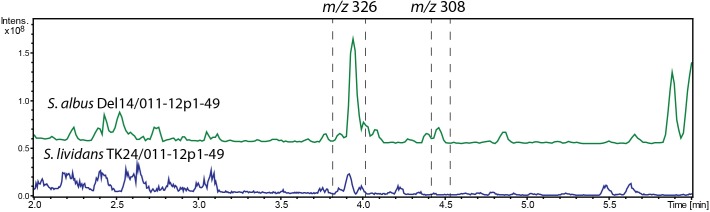
LC-MS chromatogram RT 2–6 min out of an overall 9 min gradient run of extracts of *S. albus* Del14/011-12p1-49 and *S. lividans* TK24/011-12p1-49 strains carrying the *alp*-gene cluster construct. The strains were cultivated in NL19 media and compounds were extracted from the biomass. Peaks that correspond to alpiniamides are highlighted. Alpiniamide A-B (*m/z* 326 [*M*-H_2_O+H]^+^) and alpiniamide C (*m/z* 308 [*M*-H_2_O+H]^+^).

In addition, we have cloned the 46.7 kb region of the chromosome of *Streptomyces* sp. IB2014/011-12 harboring the entire *alp* gene cluster and surrounding regions using transformation associated recombination in yeast. The cloning of the right region of *Streptomyces* sp. IB2014/011-12 chromosome and overall architecture of the cluster was verified through sequencing with MinION (Oxford Nanopore, United Kingdom). The construct was introduced into *S. lividans* TK24 and *S. albus* Del14. The recombinant strains bearing the cloned *alp*-gene cluster were found to produce alpiniamides (**Figure 5**). However, we were able to identify only alpiniamides A, B, and C in the extracts of generated strains. The lack of alpiniamide D could be caused either by a change in the behavior of the enzymatic assembly line or by the relatively low production of this minor derivative, hindering its identification. Moreover, a set of mutant constructs with deletion of *alpD*, *alpR*, and *alpE* genes were created. The resulting plasmids were introduced into *S. albus* Del14 and analyzed for production of alpiniamides. The deletion of *alpD* encoding an acyl-CoA dehydrogenase and *alpR* encoding a ribosomal RNA methyltransferase (**Table 2**) had no significant effect on the production of compounds (**Supplementary Figure S13**). On the contrary, *S. albus* Del14 carrying the construct with deleted *alpE* gene was not able to produce alpiniamides (**Supplementary Figure S13**). Lastly, in order to determine the boundaries of *alp* gene cluster we performed the *blastn* search for the actinobacterial genome lacking the *alp* gene cluster but preserving the regions flanking the cluster in the genomes of *Streptomyces* sp. IB2014/011-12. As such, the *Kitasatospora albolonga* YIM 101047 and *Streptomyces* sp. IB2014/011-12 whole genome alignment have revealed that the *alp* gene cluster include gene between *alpA1* and *alpG*, when *alpR,D* and *alpW-I* are most probably not involved in alpiniamides biosynthesis (**Supplementary Figure S14**).

### Deduction of Alpiniamide Biosynthesis

**FIGURE 6:**
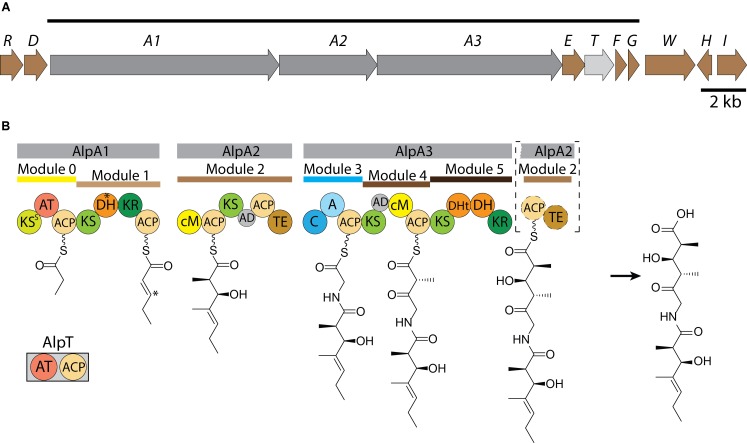
Scheme of the organization of *alp*-gene cluster **(A)**. The line above shows the deduced boundaries of the cluster. The scale bar corresponds to 2 kb. Proposed pathway for the biosynthesis of alpiniamide **(B)**. Domains: KS - ketosynthase, AT, acyltransferase; DH, dehydratase; KR, ketoreductase; ACP, acyl carrier protein; cM, C-methyltransferase; AD, acyl-transferase docking domain; TE, thioesterase; C, condensation; A, adenylation; DHt, truncated dehydratase. ^∗^Indicates the DH presumably responsible for double bond migration and corresponding position at growing alpiniamide chain.

The predicted architecture of a hybrid NRPS-trans-AT-PKS mega-enzyme encoded by the *alp* gene cluster made it possible to propose the biosynthetic steps for the assembly of alpiniamides (**Figure 6B**). Polyketide synthases lacking the acyltransferase (trans-AT-PKS) were thought to be rare systems in actinomycetes. Only recently it has been discovered that they are present in several actinobacterial strains (Helfrich and Piel, 2016). However, in general, this type of PKS enzymes is thought to be the major class of polyketide biosynthesis machineries. In these systems the usual in-line acyltransferase domains are missing, and instead, free-standing enzymes take their place (Jenner et al., 2013). Apparently, the orientation and location of the core genes in the *alp*-gene cluster do not reflect the order of the biosynthetic steps in alpiniamide assembly. The organization of the *alp* gene cluster lacks the co-linearity due to its unusual domain orders and domains acting across modules, which is a well-known feature of trans-AT-PKSs (Helfrich and Piel, 2016).

The synthesis is initiated by the loading module 0, which is part of the bimodular protein encoded by gene *alpA1* (12750) (**Table 2**). Unlike the others, this module possesses its own AT domain that is predicted to be specific for methylmalonate. Since the starter unit in alpiniamide biosynthesis is thought to be a propionate, we believe that loading module 0 loads the methylmalonate and then decarboxylates it to produce propionate. Such a decarboxylative loading process is known for several *cis*-AT-PKS systems, including the ones involved in the biosynthesis of tylosin, niddamycin, pikromycin, spinosyn, and monensin (Fouces et al., 1999; Xue and Sherman, 2001). However, unlike the cis-AT systems, which typically harbor a KS^Q^ decarboxylative domain with the active site Cys mutated to a Gln, the Cys in the canonical CHH motif of AlpA1 KS0 is substituted with a Ser (KS^S^). Similar substitutions were found in the loading KS of trans-AT oocydin synthase (Matilla et al., 2012) and several cis-AT PKSs from the nystatin, rimocidin, and pimaricin biosynthesis (Aparicio et al., 2000; Brautaset et al., 2003; Seco E.M. et al., 2004). These KS^S^ domains are proposed to participate in decarboxylation or synthesis of the starter substrates. At least in the case of the nystatin loading module, it was shown that the Ser residue of KS^S^0 is not crucial for proper initiation of the biosynthesis (Brautaset et al., 2003). However, in all of the cases described above, the polyketide chain is initiated by acetate rather than propionate. At the same time, another scenario cannot be excluded. The *alpE* gene located close to *alpA_1_* is predicted to encode the KSIII enzyme. This enzyme is similar to the daunorubicin loading KS as well as to the ACP-shuttle-type KSs from several other anthracycline polyketides that are initiated with a propionate starter unit, such as cosmomycin D and cinerubin B (Garrido et al., 2006; Kersten et al., 2013). The high similarity between AlpE and these KSs point on alternative scenario at which the decarboxylative function of the loading module in alpiniamides assembly might be provided by the standalone decarboxylative KSIII, similar to daunorubicin/aklavinone biosynthesis (Tsukamoto et al., 1992). The essentiality of AlpE for the production of alpiniamides in heterologous hosts supports this idea.

Subsequently, two elongation steps conducted by module 1 and 2 occur and incorporate two units of malonate. The substrate is passed on to the PKS by the trans-acting acyltransferase encoded by gene 12740. This protein is a bidomain AT-ACP enzyme that is predicted to be specific for malonate. The function of the ACP domain cannot be deduced from the available data, but it harbors a Ser residue for attachment of phosphopantetheine. Module 1 possesses KR and DH domains for ketoreduction and dehydration reactions, respectively. As expected, a double bond is present in alpiniamide A, C, and D between C-4 and C-5. However, in the case of alpiniamides B_1_ and B_2_, the DH domain is apparently skipped, resulting in a hydroxyl group at C-5. The ketoreductase domain is missing in module 2; although, alpiniamides have the hydroxyl group at the corresponding position. A possible explanation for this would be that the activity of the KR domain from module 1 is shared between modules 1 and 2. Interestingly B_1_ and B_2_ differ in the stereochemistry at C-5 position. Ketoreductases are highly stereospecific and are divided into groups depending on which stereochemistry they provide (Keatinge-Clay, 2016). The *in silico* analysis of module 1 KR place it in B1 stereospecificity type performing D-α-reduction that correspond to the configuration observed in alpiniamide B_2_ (Kitsche and Kalesse, 2013). Therefore, it is unclear how the different stereochemistry at position C-5 as well as the *threo* configuration at C-2/C-3 is generated. Module 2 has a C-methyltransferase domain. The acyltransferases in trans-AT-PKS are known to solely utilize malonyl-CoA as opposed to branched substrates as elongation units, and the side chain methyl groups are typically incorporated by C-methyltransferases. This seems to also be true in the case of the alpiniamides. From the labeled methionine feeding experiment, we clearly see that the methyl groups of the alpiniamides are introduced by the S-adenosyl-methionine (SAM) dependent methyltransferases present in module 2 and 4. Since these compounds each contain four methyl groups, we assume that both methyltransferases need to act twice during the biosynthesis. Thus, modules 2 and 4 provide the C-methyltransferase activity also to module 1 and 5. Another key feature of module 2 is the presence of a thioesterase domain. This TE is predicted to be a class II TE of the α/β hydrolase family. These enzymes hydrolyze the residues attached to the phosphopantetheine group of ACPs. The exact function of the TE domain of AlpA2 is not clear, especially considering its location. A similar architecture was observed in the case of the burkholderic acid trans-AT PKS, which harbors internal TE domains with unknown functions (Franke et al., 2012). TE II domains are often not essential for biosynthesis, but can be involved in removing aberrant substrates from stalled PKS megasynthases and control the loading of the starter unit (Kotowska and Pawlik, 2014). The TE domain of AlpA2 has the Cys in place of Ser residue of catalytic triad Ser/Asp/His (**Supplementary Figure S15**). However, such substitutions are not rare and can be found in TE14 family of thioesterases (Cantu et al., 2010).

Another interesting feature of module 2 is the possible migration of the double bond. The double bond in alpiniamides B_1_ and B_2_ is shifted from the canonical position between C-2 and C-3 to the position between C-3 and C-4. A so-called double bond migration is described for several trans-AT produced polyketides such as bacillaene, corallopyronin A, and rhizoxin (Butcher et al., 2007; Partida-Martinez and Hertweck, 2007; Lohr et al., 2013), and this process is thought to be mediated by a dehydratase domain either by direct β-γ dehydration or α-β dehydration and subsequent isomerization (Gay et al., 2014). However, it is not clear if alpiniamides B_1_ and B_2_ arise from alpiniamide A through the catalytic action of one of the DH domains (most likely by the DHt of module 5 that is predicted to be non-functional) or from an acid-induced double bond migration.

At the next step, a non-ribosomal peptide synthetase module within AlpA_3_ extend the polyketide intermediate with a glycine. Interestingly, glycine is often found to act as a bridge between two polyketide chains in hybrid NRPS-trans-AT-PKS assembled natural products, such as bacillaene, batumin, calyculin, corallopyronin, misakinolide and oxazolomycin (Helfrich and Piel, 2016).

Modules 4 and 5 further extend the molecule with two malonate units. Typically, ketosynthases ensuing a NRPS module are highly substrate specific and do rarely accept aberrant substrates with bulkier amino acid residues (Kohlhaas et al., 2013). As in module 2, module 4 lacks the ketoreductive function even though the hydroxy group at C-3′ is present. This activity is most likely provided by module 5, which contains KR and DH domains. However, based on the structures of alpiniamide A, B and D, we assume that the DH domain is occasionally skipped. Only alpiniamide C seems to be dehydrated at this step, which resulted in the double bond between C-3′ and C-4′. Module 5 is missing the ACP and TE domains, which are instead located in module 2. The mechanism by which alpiniamides are released from the biosynthetic enzyme is not clear. Most likely, the TE activity is provided by the respective domain of module 2. However, the location of the TE domain is unusual as well as the composition of catalytic triad (Cys/Asp/His). The fact that it is placed right before the NRPS module 3 makes us think that it also might be involved in the cleavage of aberrant substrates than in the release of the final product.

In the case of alpiniamide D, module 2 is probably skipped entirely during the biosynthesis since it lacks the C-1 and C-2 part of the molecule. Such a case is very rare but was previously described for epothilone K (Moss et al., 2004) and for the trans-AT-PKS-derived compound albicidin (Huang et al., 2001).

Even though alpiniamides are quite small molecules they provide a fascinating example of high chemical diversity introduced by the PKS assembly line. The alpiniamides NRPS-trans-AT-PKS utilizes a combination of domain and module skipping, in-line methylation and double bond migration events to produce a variety of chemical structures from a simple initial building block.

## Conclusion

We have isolated five new alpiniamide derivatives and identified the gene cluster responsible for their biosynthesis in the new *Streptomyces* sp. IB2014/011-12. This shows that there are still great opportunities to discover promising natural products, especially from unique and largely unexplored ecological niches such as Lake Baikal.

## Author Contributions

CP, YR, and JZ performed the experiments and analyzed the data. CR and JK sequenced and assembled the genome. AL conceived and designed the experiments. All authors participated in the manuscript preparation and discussion.

## Conflict of Interest Statement

The authors declare that the research was conducted in the absence of any commercial or financial relationships that could be construed as a potential conflict of interest.The reviewer YL and handling Editor declared their shared affiliation.

## References

[B1] Allam N. G. (2012). Protective role of *Aspergillus fumigatus* melanin against ultraviolet (UV) irradiation and *Bjerkandera adusta* melanin as a candidate vaccine against systemic candidiasis. *Afr. J. Biotechnol.* 11 6566–6577. 10.5897/AJB11.4136

[B2] Aparicio J. F. Fouces R. Mendes M. V. Olivera N. Martín J. F. (2000). A complex multienzyme system encoded by five polyketide synthase genes is involved in the biosynthesis of the 26-membered polyene macrolide pimaricin in *Streptomyces natalensis*. *Chem. Biol.* 7 895–905. 10.1016/S1074-5521(00)00038-7 11094342

[B3] Axenov-Gribanov D. Rebets Y. Tokovenko B. Voytsekhovskaya I. Timofeyev M. Luzhetskyy A. (2016). The isolation and characterization of actinobacteria from dominant benthic macroinvertebrates endemic to Lake Baikal. *Folia Microbiol.* 61 159–168. 10.1007/s12223-015-0421-z 26347255

[B4] Barona-Gómez F. Wong U. Giannakopulos A. E. Derrick P. J. Challis G. L. (2004). Identification of a cluster of genes that directs desferrioxamine biosynthesis in *Streptomyces coelicolor* M145. *J. Am. Chem. Soc.* 126 16282–16283. 10.1021/ja045774k 15600304

[B5] Bifulco G. Dambruoso P. Gomez-Paloma L. Riccio R. (2007). Determination of relative configuration in organic compounds by NMR spectroscopy and computational methods. *Chem. Rev.* 107 3744–3779. 10.1021/cr030733c 17649982

[B6] Bilyk O. Sekurova O. N. Zotchev S. B. Luzhetskyy A. (2016). Cloning and heterologous expression of the grecocycline biosynthetic gene cluster. *PLoS One* 11:e0158682. 10.1371/journal.pone.0158682 27410036 PMC4943663

[B7] Blodgett J. A. V. Thomas P. M. Li G. Velasquez J. E. van der Donk W. A. Kelleher N. L. (2007). Unusual transformations in the biosynthesis of the antibiotic phosphinothricin tripeptide. *Nat. Chem. Biol.* 3 480–485. 10.1038/nchembio.2007.9 17632514 PMC4313788

[B8] Brautaset T. Borgos S. E. F. Sletta H. Ellingsen T. E. Zotchev S. B. (2003). Site-specific mutagenesis and domain substitutions in the loading module of the nystatin polyketide synthase, and their effects on nystatin biosynthesis in *Streptomyces noursei*. *J. Biol. Chem.* 278 14913–14919. 10.1074/jbc.M212611200 12594224

[B9] Butcher R. A. Schroeder F. C. Fischbach M. A. Straight P. D. Kolter R. Walsh C. T. (2007). The identification of bacillaene, the product of the PksX megacomplex in *Bacillus subtilis*. *Proc. Natl. Acad. Sci. U.S.A.* 104 1506–1509. 10.1073/pnas.0610503104 17234808 PMC1785240

[B10] Cantu D. C. Chen Y. Reilly P. J. (2010). Thioesterases: a new perspective based on their primary and tertiary structures. *Protein Sci.* 19 1281–1295. 10.1002/pro.417 20506386 PMC2974821

[B11] Cao S. Blodgett J. A. V. Clardy J. (2010). Targeted discovery of polycyclic tetramate macrolactams from an environmental *Streptomyces* strain. *Org. Lett.* 12 4652–4654. 10.1021/ol1020064 20843016 PMC2952660

[B12] Dale J. A. Mosher H. S. (1973). Nuclear magnetic resonance enantiomer regents. Configurational correlations via nuclear magnetic resonance chemical shifts of diastereomeric mandelate, O-methylmandelate, and.alpha.-methoxy-.alpha.-trifluoromethylphenylacetate (MTPA) esters. *J. Am. Chem. Soc.* 95 512–519. 10.1021/ja00783a034

[B13] Dias D. A. Urban S. Roessner U. (2012). A historical overview of natural products in drug discovery. *Metabolites* 2 303–336. 10.3390/metabo2020303 24957513 PMC3901206

[B14] Ding Y. Li Y. Li Z. Zhang J. Lu C. Wang H. (2016). Alteramide B is a microtubule antagonist of inhibiting *Candida albicans*. *Biochim. Biophys. Acta* 1860 2097–2106. 10.1016/j.bbagen.2016.06.025 27373684 PMC4961524

[B15] Fouces R. Mellado E. Díez B. Barredo J. L. (1999). The tylosin biosynthetic cluster from *Streptomyces fradiae*: genetic organization of the left region. *Microbiology* 145(Pt 4), 855–868. 10.1099/13500872-145-4-855 10220165

[B16] Franke J. Ishida K. Hertweck C. (2012). Genomics-driven discovery of burkholderic acid, a noncanonical, cryptic polyketide from human pathogenic *Burkholderia* species. *Angew. Chem.* 51 11611–11615. 10.1002/anie.201205566 23055407

[B17] Garrido L. M. Lombó F. Baig I. Nur-E-Alam M. Furlan R. L. A. Borda C. C. (2006). Insights in the glycosylation steps during biosynthesis of the antitumor anthracycline cosmomycin: characterization of two glycosyltransferase genes. *Appl. Microbiol. Biotechnol.* 73 122–131. 10.1007/s00253-006-0453-z 16810496 PMC2879347

[B18] Gay D. C. Spear P. J. Keatinge-Clay A. T. (2014). A double-hotdog with a new trick: structure and mechanism of the trans-acyltransferase polyketide synthase enoyl-isomerase. *ACS Chem. Biol.* 9 2374–2381. 10.1021/cb500459b 25089587 PMC4201341

[B19] Gietz R. D. Schiestl R. H. (2007). High-efficiency yeast transformation using the LiAc/SS carrier DNA/PEG method. *Nat. Protoc.* 2 31–34. 10.1038/nprot.2007.13 17401334

[B20] Golinska P. Wypij M. Agarkar G. Rathod D. Dahm H. Rai M. (2015). Endophytic actinobacteria of medicinal plants: diversity and bioactivity. *Antonie Van Leeuwenhoek* 108 267–289. 10.1007/s10482-015-0502-7 26093915 PMC4491368

[B21] Green M. R. Sambrook J. (2012). *Molecular Cloning: A Laboratory Manual.* Cold Spring Harbor, NY: Cold Spring Harbor Laboratory Press.

[B22] Gust B. Chandra G. Jakimowicz D. Yuqing T. Bruton C. J. Chater K. F. (2004). *λ Red-Mediated Genetic Manipulation of Antibiotic-Producing Streptomyces.* New York, NY: Elsevier, 107–128. 10.1016/S0065-2164(04)54004-2 15251278

[B23] Hasani A. Kariminik A. Issazadeh K. (2014). Streptomycetes: characteristics and their antimicrobial activities. *IJABBR* 2 63–75.

[B24] Helfrich E. J. N. Piel J. (2016). Biosynthesis of polyketides by trans-AT polyketide synthases. *Nat. Prod. Rep.* 33 231–316. 10.1039/c5np00125k 26689670

[B25] House H. O. Crumrine D. S. Teranishi A. Y. Olmstead H. D. (1973). Chemistry of carbanions. XXIII. Use of metal complexes to control the aldol condensation. *J. Am. Chem. Soc.* 95 3310–3324. 10.1021/ja00791a039

[B26] Hoye T. R. Jeffrey C. S. Shao F. (2007). Mosher ester analysis for the determination of absolute configuration of stereogenic (chiral) carbinol carbons. *Nat. Protoc.* 2 2451–2458. 10.1038/nprot.2007.354 17947986

[B27] Huang G. Zhang L. Birch R. G. (2001). A multifunctional polyketide-peptide synthetase essential for albicidin biosynthesis in *Xanthomonas albilineans*. *Microbiology* 147 631–642. 10.1099/00221287-147-3-631 11238970

[B28] Jakobi M. Winkelmann G. Kaiser D. Kempter C. Jung G. Berg G. (1996). Maltophilin: a new antifungal compound produced by *Stenotrophomonas maltophilia* R3089. *J. Antibiot.* 49 1101–1104. 10.7164/antibiotics.49.1101 8982338

[B29] Jenner M. Frank S. Kampa A. Kohlhaas C. Pöplau P. Briggs G. S. (2013). Substrate specificity in ketosynthase domains from trans-AT polyketide synthases. *Angew. Chem.* 52 1143–1147. 10.1002/anie.201207690 23212972

[B30] Keatinge-Clay A. T. (2016). Stereocontrol within polyketide assembly lines. *Nat. Prod. Rep.* 33 141–149. 10.1039/c5np00092k 26584443 PMC4742408

[B31] Kersten R. D. Ziemert N. Gonzalez D. J. Duggan B. M. Nizet V. Dorrestein P. C. (2013). Glycogenomics as a mass spectrometry-guided genome-mining method for microbial glycosylated molecules. *Proc. Natl. Acad. Sci. U.S.A.* 110 E4407–E4416. 10.1073/pnas.1315492110 24191063 PMC3839717

[B32] Khosla C. (2009). Structures and mechanisms of polyketide synthases. *J. Org. Chem.* 74 6416–6420. 10.1021/jo9012089 19711990

[B33] Kieser T. (2000). *Practical Streptomyces Genetics.* Norwich: Innes.

[B34] Kitsche A. Kalesse M. (2013). Configurational assignment of secondary hydroxyl groups and methyl branches in polyketide natural products through bioinformatic analysis of the ketoreductase domain. *Chembiochem* 14 851–861. 10.1002/cbic.201300063 23576424

[B35] Kohlhaas C. Jenner M. Kampa A. Briggs G. S. Afonso J. P. Piel J. (2013). Amino acid-accepting ketosynthase domain from a trans-AT polyketide synthase exhibits high selectivity for predicted intermediate. *Chem. Sci.* 4 3212–3217. 10.1039/c3sc50540e

[B36] Kotowska M. Pawlik K. (2014). Roles of type II thioesterases and their application for secondary metabolite yield improvement. *Appl. Microbiol. Biotechnol.* 98 7735–7746. 10.1007/s00253-014-5952-8 25081554 PMC4147253

[B37] Latypov S. K. Seco J. M. Quiñoá E. Riguera R. (1996). MTPA vs MPA in the determination of the absolute configuration of chiral alcohols by 1 H NMR. *J. Org. Chem.* 61 8569–8577. 10.1021/jo960719i

[B38] Li S. Jochum C. C. Yu F. Zaleta-Rivera K. Du L. Harris S. D. (2008). An antibiotic complex from *Lysobacter enzymogenes* strain C3: antimicrobial activity and role in plant disease control. *Phytopathology* 98 695–701. 10.1094/PHYTO-98-6-0695 18944294

[B39] Lohr F. Jenniches I. Frizler M. Meehan M. J. Sylvester M. Schmitz A. (2013). α,β → β,γ double bond migration in corallopyronin A biosynthesis. *Chem. Sci.* 4 4175–4180. 10.1039/C3SC51854J

[B40] Lou L. Qian G. Xie Y. Hang J. Chen H. Zaleta-Rivera K. (2011). Biosynthesis of HSAF, a tetramic acid-containing macrolactam from *Lysobacter enzymogenes*. *J. Am. Chem. Soc.* 133 643–645. 10.1021/ja105732c 21171605 PMC3078565

[B41] Makar A. B. McMartin K. E. Palese M. Tephly T. R. (1975). Formate assay in body fluids: application in methanol poisoning. *Biochem. Med.* 13 117–126. 10.1016/0006-2944(75)90147-7 1

[B42] Mansjö M. Johansson J. (2011). The riboflavin analog roseoflavin targets an FMN-riboswitch and blocks *Listeria monocytogenes* growth, but also stimulates virulence gene-expression and infection. *RNA Biol.* 8 674–680. 10.4161/rna.8.4.15586 21593602 PMC3225981

[B43] Matilla M. A. Stöckmann H. Leeper F. J. Salmond G. P. C. (2012). Bacterial biosynthetic gene clusters encoding the anti-cancer haterumalide class of molecules: biogenesis of the broad spectrum antifungal and anti-oomycete compound, oocydin A. *J. Biol. Chem.* 287 39125–39138. 10.1074/jbc.M112.401026 23012376 PMC3493953

[B44] Matsumori N. Kaneno D. Murata M. Nakamura H. Tachibana K. (1999). Stereochemical determination of acyclic structures based on carbon-proton spin-coupling constants. A method of configuration analysis for natural products. *J. Org. Chem.* 64 866–876. 10.1021/jo981810k 11674159

[B45] Meyer F. Goesmann A. McHardy A. C. Bartels D. Bekel T. Clausen J. (2003). GenDB–an open source genome annotation system for prokaryote genomes. *Nucleic Acids Res.* 31 2187–2195. 10.1093/nar/gkg312 12682369 PMC153740

[B46] Moss S. J. Martin C. J. Wilkinson B. (2004). Loss of co-linearity by modular polyketide synthases: a mechanism for the evolution of chemical diversity. *Nat. Prod. Rep.* 21 575–593. 10.1039/b315020h 15459756

[B47] Myronovskyi M. Welle E. Fedorenko V. Luzhetskyy A. (2011). Beta-glucuronidase as a sensitive and versatile reporter in actinomycetes. *Appl. Environ. Microbiol.* 77 5370–5383. 10.1128/AEM.00434-11 21685164 PMC3147471

[B48] Palecková P. Bobek J. Mikulík K. (2009). tmRNA of *Streptomyces collinus* and *Streptomyces griseus* during the growth and in the presence of antibiotics. *Microb. Biotechnol.* 2 114–122. 10.1111/j.1751-7915.2008.00066.x 21261886 PMC3815426

[B49] Partida-Martinez L. P. Hertweck C. (2007). A gene cluster encoding rhizoxin biosynthesis in “*Burkholderia rhizoxina*”, the bacterial endosymbiont of the fungus *Rhizopus microsporus*. *Chembiochem* 8 41–45. 10.1002/cbic.200600393 17154220

[B50] Procópio R. E. Silva I. R. Martins M. K. Azevedo J. L. Araújo J. M. (2012). Antibiotics produced by Streptomyces. *Braz. J. Infect. Dis.* 16 466–471. 10.1016/j.bjid.2012.08.014 22975171

[B51] Running W. (1993). Computer software reviews. Chapman and hall dictionary of natural products on CD-ROM. *J. Chem. Inf. Model.* 33 934–935. 10.1021/ci00016a603

[B52] Schwarz J. Konjik V. Jankowitsch F. Sandhoff R. Mack M. (2016). Identification of the key enzyme of roseoflavin biosynthesis. *Angew. Chem.* 55 6103–6106. 10.1002/anie.201600581 27062037

[B53] Seco E. M. Pérez-Zúñiga F. J. Rolón M. S. Malpartida F. (2004). Starter unit choice determines the production of two tetraene macrolides, rimocidin and CE-108, in Streptomyces diastaticus var. 108. *Chem. Biol.* 11 357–366. 10.1016/j.chembiol.2004.02.017 15123265

[B54] Seco J. M. Quiñoá E. Riguera R. (2004). The assignment of absolute configuration by NMR †. *Chem. Rev.* 104 17–118. 10.1021/cr000665j 22658125

[B55] Seemann T. (2014). Prokka: rapid prokaryotic genome annotation. *Bioinformatics* 30 2068–2069. 10.1093/bioinformatics/btu153 24642063

[B56] Shigemori H. Bae M. A. Yazawa K. Sasaki T. Kobayashi J. (1992). Alteramide A, a new tetracyclic alkaloid from a bacterium *Alteromonas* sp. associated with the marine sponge *Halichondria okadai*. *J. Org. Chem.* 57 4317–4320. 10.1021/jo00041a053

[B57] Stiles M. Winkler R. R. Chang Y.-L. Traynor L. (1964). Stereochemical assignments for β-Ketols formed by aldol addition of three simple ketones to p-nitrobenzaldehyde. *J. Am. Chem. Soc.* 86 3337–3342. 10.1021/ja01070a027

[B58] Tsukamoto N. Fujii I. Ebizuka Y. Sankawa U. (1992). Cloning of aklavinone biosynthesis genes from *Streptomyces galilaeus*. *J. Antibiot.* 45 1286–1294. 10.7164/antibiotics.45.1286 1399850

[B59] Ueda K. Oinuma K.-I. Ikeda G. Hosono K. Ohnishi Y. Horinouchi S. (2002). AmfS, an extracellular peptidic morphogen in *Streptomyces griseus*. *J. Bacteriol.* 184 1488–1492. 10.1128/JB.184.5.1488-1492.2002 11844785 PMC134859

[B60] Vaishnav P. Demain A. L. (2011). Unexpected applications of secondary metabolites. *Biotechnol. Adv.* 29 223–229. 10.1016/j.biotechadv.2010.11.006 21130862

[B61] Ventola C. L. (2015). The antibiotic resistance crisis: part 1: causes and threats. *Pharm. Ther.* 40 277–283. 25859123 PMC4378521

[B62] Weber T. Blin K. Duddela S. Krug D. Kim H. U. Bruccoleri R. (2015). antiSMASH 3.0-a comprehensive resource for the genome mining of biosynthetic gene clusters. *Nucleic Acids Res.* 43 W237–W243. 10.1093/nar/gkv437 25948579 PMC4489286

[B63] Whittle M. Willett P. Klaffke W. van Noort P. (2003). Evaluation of similarity measures for searching the dictionary of natural products database. *J. Chem. Inf. Comput. Sci.* 43 449–457. 10.1021/ci025591m 12653508

[B64] Winston F. Dollard C. Ricupero-Hovasse S. L. (1995). Construction of a set of convenient *Saccharomyces cerevisiae* strains that are isogenic to S288C. *Yeast* 11 53–55. 10.1002/yea.320110107 7762301

[B65] Woo P. C. Y. Tam E. W. T. Chong K. T. K. Cai J. J. Tung E. T. K. Ngan A. H. Y. (2010). High diversity of polyketide synthase genes and the melanin biosynthesis gene cluster in *Penicillium marneffei*. *FEBS J.* 277 3750–3758. 10.1111/j.1742-4658.2010.07776.x 20718860

[B66] Xu K. Yang P.-F. Yang Y.-N. Feng Z.-M. Jiang J.-S. Zhang P.-C. (2017). Direct assignment of the *Threo* and *Erythro* configurations in polyacetylene glycosides by 1H NMR spectroscopy. *Org. Lett.* 19 686–689. 10.1021/acs.orglett.6b03855 28102685

[B67] Xu L. Wu P. Wright S. J. Du L. Wei X. (2015). Bioactive polycyclic tetramate macrolactams from *Lysobacter enzymogenes* and their absolute configurations by theoretical ECD calculations. *J. Nat. Prod.* 78 1841–1847. 10.1021/acs.jnatprod.5b00099 26200218

[B68] Xue Y. Sherman D. H. (2001). Biosynthesis and combinatorial biosynthesis of pikromycin-related macrolides in *Streptomyces venezuelae*. *Metab. Eng.* 3 15–26. 10.1006/mben.2000.0167 11162229

[B69] Zhou H. Yang Y. Zhang J. Peng T. Zhao L. Xu L. (2013). Alkaloids from an endophytic *Streptomyces* sp. YIM66017. *Nat. Prod. Commun.* 8 1393–1396. 24354182

[B70] Zhu D. Liu J. Han R. Shen G. Long Q. Wei X. (2014). Identification and characterization of ectoine biosynthesis genes and heterologous expression of the ectABC gene cluster from *Halomonas* sp. QHL1, a moderately halophilic bacterium isolated from Qinghai Lake. *J. Microbiol.* 52 139–147. 10.1007/s12275-014-3389-5 24500478

