# Supplementary material for: New Alpiniamides From Streptomyces sp. IB2014/011-12 Assembled by an Unusual Hybrid Non-ribosomal Peptide Synthetase Trans-AT Polyketide Synthase Enzyme
Source: Front Microbiol. 2018 Aug 22;9:1959. doi: 10.3389/fmicb.2018.01959 (PMC6113372; doi:10.3389/fmicb.2018.01959)
Supplement: Supplementary file 1 [file Data_Sheet_1.docx]

Supplementary Material

New Alpiniamides from *Streptomycetes sp.* 11-12 assembled by an unusual hybrid-nonribosomal peptide synthetase-transAT-polyketidesynthase enzyme

Constanze Paulus ^1^, Yuriy Rebets ^2^, Josef Zapp^2^, Christian Rückert^3^, Jörn Kalinowski^3^, Andriy Luzhetskyy ^1, 2,^*

^1^ Helmholtz-Institute for Pharmaceutical Research, Saarbrücken, Germany

^2^ Department for pharmaceutical Biotechnology, University of Saarland, Saarbrücken, Germany

^3^ Center for Biotechnology (CeBiTec), Bielefeld University, Bielefeld, Germany

# Supplementary Data

Supplementary Material should be uploaded separately on submission. Please include any supplementary data, figures and/or tables.

# Supplementary Figures and Tables

## Supplementary tables

**Table S1.** Primer used in this work.

| Name | sequence |
| --- | --- |
| 11-12_12720ChF | TCGACAGACAGCTGCCCTG |
| 11-12_12720ChR | GCACACAGTAGAACGTGTTG |
| 11-12_12715ChF | ACCCGAAGTTCGTGGTCCTG |
| 11-12_12715ChR | CGGACCGTTGCGTGTTCACA |
| 11-12_12695ChF | CACGCTGGTGTTCGACTACC |
| 11-12_12695ChR | AGAGCTCTTCGATGGGGTAG |
| 11-12_12720DF | TGGGGCGCCCCCGGTTCCTCGGTTAGCCTGAGTGACATATGAAACTACGCCCCCAACTGAGAG |
| 11-12_12720DR | GGAAGGGCCGGGAACCCTGATCTCCGCCGTCAGCGCTTGACTCGACCCGGTACCGGAGTA |
| 11-12_12715DF | CACGGGCGACGCAGACGCGAGACTCTGGAGGGGCCGTGCAACTACGCCCCCAACTGAGAG |
| 11-12_12715DR | GACGCGTCGCGCCACCGCGAGCCAGCGCCTCACCGCCGCACTCGACCCGGTACCGGAGTA |
| 11-12_12695DF | TGAGAGGGAACGAGAAGTGAAGACAGAGGCCCTGTTCATCGACTACGCCCCCAACTGAGAG |
| 11-12_12695DR | CCCTGTCCCGGGAACACGTAGCAGCGCATCAGAGCCGCCCGCTCGACCCGGTACCGGAGTA |
| C9-2REcV | atGATATCATCCAGGAGATGATCTCGCG |
| C9-2FEcI | atGAATTCAATCTGGACCGCAACGGCCT |
| C9-1FEcV | atGATATCG CCTGAAGGACCAACTCGT |
| C9-1RXba | atTCTAGAACTTCGATGGGAT CGCCGA |
| 11-12DelCheckF | AAGTGCTGGTCGAGGACGTA |
| 11-12DelCheckR | TACGCCTGGCTGATGG AGAT |
| 11-12C9TarF1Not | ATGCGGCCGCAACGGCGTGAAGCCGAAGAT |
| 11-12C9TarR1Nhe | ATGCTAGCGAATGCTGCTCATGGGGTGT |
| 11-12C9TarF2NheI | ATGCTAGCCTGCACACGATCATCAGCGA |
| 11-12C9TarR2HindIII | ATAAGCTTGATTGGACGGAATGTCCGTG |
| 11-12C9CheckHind | GACGAC ACGTACTACCAGGA |
| pCLYCheckHind | CTCTTCGGTGAACTTCGGG |

Sequence highlighted in yellow anneal with the priming sites of hygromycin resistance cassette from patt-shyg-oriT.

Table S2. Secondary metabolism gene clusters identified within the genome of *Streptomyces sp.* IB2014/011-12 with the use of the antiSMASH software.

| BGC | Type | Most similar known cluster. |
| --- | --- | --- |
| 1 | Lantipeptide | AmfS antibiotic (Ueda et al., 2002) |
| 2 | Terpene | - |
| 3 | Lassopeptide | Lassopetide similar to SRO15-2005 (Kersten et al., 2011) |
| 4 | Lantipeptide | - |
| 5 | Siderophore | Desferrioxamine B (Barona-Gómez et al., 2004) |
| 6 | Bacteriocin | - |
| 7 | Butyrolactone | - |
| 8 | Terpene | - |
| 9 | NRPS-Trans-at-type 1-PKS | - |
| 10 | Melanin-NRPS | Peptide similar to Coelichelin (Challis and Ravel, 2000) |
| 11 | Type 3 PKS | - |
| 12 | NRPS | - |
| 13 | type 1 PKS-NRPS | Similar to polycyclic tetramate macrolactams (Luo et al., 2013) |
| 14 | Terpene | Squalene antibiotic similar to Hopene (Pan et al., 2015) |
| 16 | Type 1 PKS | - |
| 17 | Other | Riboflavin antibiotic similar to Roseoflavin (Schwarz et al., 2016) |
| 18 | Terpene | - |
| 19 | thiopeptide | - |
| 20 | Melanin | Phenoxazinone antibiotic Grixazone (Suzuki et al., 2007) |
| 21 | Ectoine | Ectoine (Zhu et al., 2014) |
| 22 | NRPS | - |
| 23 | Type 3 PKS | Alkylresorcinol (Cook et al., 2010) |
| 24 | Melanin | Dihydroxynaphthalene Melanin (Woo et al., 2010) |
| 25 | NRPS | - |
| 26 | Siderophore | - |
| 27 | Type 2 PKS | - |
| 28 | Terpene | - |
| 29 | NRPS | - |

**Table S3.** NMR spectroscopic data (MeOD_4_) for alpiniamides 1-5.

| **Position** | **(1)** | | **(1)^1^** | | **(2)** | | **(3)** | | **(4)** | | **(5)** | |
| --- | --- | --- | --- | --- | --- | --- | --- | --- | --- | --- | --- | --- |
|  | **δ_C_ ^a^** | δ_H_ ^b^ | δ_C_ ^a^ | **δ_H_ ^b^** | **δ_C_ ^a^** | δ_H_ ^b^ | δ_C_ ^a^ | δ_H_ ^b^ | **δ_C_ ^a^** | **δ_H_ ^b^** | **δ_C_ ^a^** | **δ_H_ ^b^** |
| 1 | 178.3, C |  | 176.75, C |  | 178.0, C |  | 177.2, C |  | 178.2, C |  | 172.2, C |  |
| 2 | 45.1, CH | 2.54, dq (9.5, 7.0) | 44.28, CH | 2.52, dq (9.5, 7.0) | 40.4, CH | 3.36, dq (9.5, 7.0) | 40.5, CH | 3.36, dq (9.5, 7.0) | 45.5, CH | 2.56, dq (9.5, 7.1) | 130.7, C |  |
| 3 | 81.0, CH | 3.98, d (9.5) | 80.40, CH | 4.03, d (9.5) | 126.6, CH | 5.43, br d (9.3) | 127.2, CH | 5.43, br d (9.3) | 81.0, CH | 3.98, d (9.5) | 139.4, CH | 6.39, tq (7.2, 1.5) |
| 4 | 135.1, C |  | 133.13, C |  | 140.6, C |  | 139.8, C |  | 135.0, C |  | 22.3, C | 2.20, quint (7.5, 1.0) |
| 5 | 131.8, CH | 5.41, t (7.2) | 131.94, CH | 5.40, t (7.2) | 79.3, CH | 3.85, t (6.7) | 79.8, CH | 3.85, t (6.8) | 132.0, CH | 5.42, t (7.3) | 13.2, CH_3_ | 1.05, t (7.5) |
| 6 | 21.5, CH_2_ | 2.06, quint (7.5) | 20.85, CH_2_ | 2.02, quint (7.5) | 28.5, CH_2_ | 1.54, m | 28.1, CH_2_ | 1.55, m | 21.5, CH_2_ | 2.06, quint (7.5) | 12.2, CH_3_ | 1.85, br s |
| 7 | 14.1, CH_3_ | 0.97, t (7.5) | 13.88, CH_3_ | 0.94, t (7.5) | 10.1, CH_3_ | 0.85, t (7.5) | 10.3, CH_3_ | 0.85, t (7.5) | 14.2, CH_3_ | 0.97, t (7.5) |  |  |
| 8 | 14.8, CH_3_ | 0.93, d (7.0) | 14.38, CH_3_ | 0.95, d (7.0) | 18.5, CH_3_ | 1.22, d (7.0) | 18.6, CH_3_ | 1.20, d (7.0) | 14.7, CH_3_ | 0.94, d (7.0) |  |  |
| 9 | 10.3, CH_3_ | 1.60, s | 10.41, CH_3_ | 1.57, s | 11.6, CH_3_ | 1.66, d (1.5) | 11.1, CH_3_ | 1.66, d (1.5) | 10.5, CH_3_ | 1.61, s |  |  |
| 1’ | 178.4, C |  | 179.03, C |  | 178.7, C |  | 178.3, C |  | 180.8, C |  | 178.1, C |  |
| 2’ | 44.6, CH | 2.50, quint (7.0) | 42.71, CH | 2.54, quint (7.0) | 44.60, CH | 2.48, quint (7.0) | 44.7, CH | 2.47, quint (7.0) | 44.3, CH | 3.41, dq (9.5, 7.1) | 44.4, CH | 2.55, quint (7.1) |
| 3’ | 74.2, CH | 4.01, ddd (7.0, 5.3, 1.5) | 73.54; CH | 4.02, m | 74.4, CH | 3.96, ddd (7.0, 5.3, 1.5) | 74.4, CH | 3.95, ddd (7.0, 5.3, 1.5) | 146.7, CH | 6.87, dq (9.5, 1.5) | 74.1, CH | 4.03, dd (7.1, 5.3) |
| 4’ | 47.7, CH | 2.92, qd (7.0, 5.3) | 47.23, CH | 2.79, d (7.0, 4.5) | 47.9, CH | 2.87, qd (7.0, 5.5) | 48.1, CH | 2.88, qd (7.0, 5.5) | 135.0, CH |  | 47.7, CH | 2.93, qd (7.1, 5.3) |
| 5’ | 209.9, C |  | 209.27, C |  | 209.5, C |  | 209.1, C |  | 197.4, C |  | 209.9, C |  |
| 6’ | 49.0, CH_2_ | 4.13, d (18.5)  4.25, d (18.5) | 48.81, CH_2_ | 4.19, dd (19.0, 5.0)  4.29, dd (19.0, 5.5) | 48.8 CH_2_ | 4.12, d (18.5)  4.16, d (18.5) | 48.8, CH_2_ | 4.13, d (18.5)  4.17, d (18.5) | 46.0, CH_2_ | 4.44, d (18.5)  4.48, d (18.5) | 49.0, CH_2_ | 4.18, d (18.5)  4.22, d (18.5) |
| 7’ | 15.0, CH_3_ | 1.16, d (7.0) | 14.49, CH_3_ | 1.17, d (7.0) | 14.9, CH_3_ | 1.15, d (7.0) | 15.0, CH_3_ | 1.15, d (7.0) | 18.4, CH_3_ | 1.30, d (7.0) | 14.8, CH_3_ | 1.15, d (7.0) |
| 8’ | 10.5, CH_3_ | 1.13, d (7.0) | 9.79, CH_3_ | 1.14, d (7.0) | 10.8, CH_3_ | 1.12, d (7.0) | 10.8, CH_3_ | 1.12, d (7.0) | 11.2, CH_3_ | 1.82, d (1.5) | 10.6, CH_3_ | 1.14, d (7.0) |
| NH |  |  |  | 7.09, t (5.3) |  |  |  |  |  |  |  |  |

^1^ = NMR data aquired in CDCl_3_; ^a^ = followed by multiplicity; ^b^ = followed by coupling constant J in Hz; all ^13^C chemical shifts were taken from 2D spectra HSQC/HMBC except for (1) in CDCl_3_.

**Table S4.** ^1^H chemical shifts (in ppm, recorded in CDCl_3_) and ∆δ_(S-R)_ values for Mosher derivatives 6R and 6S of alpiniamide A (**1**), 7R and 7S of alpiniamide B_1_ (**2**) and 8R and 8S of alpiniamide B_2_ (**3**).

| Position | **(1)** | | | **(2)** | | | **(3)** | | | |
| --- | --- | --- | --- | --- | --- | --- | --- | --- | --- | --- |
|  | 6S **δ_H_** | 6R **δ_H_** | ∆δ_(S-R)_ | 7S **δ_H_** | 7R **δ_H_** | ∆δ_(S-R)_ | 8S **δ_H_** | | 8R **δ_H_** | ∆δ_(S-R)_ |
| 1 | - | - | - | - | - | - | - | - | | - |
| 2 | 2.69 | 2.72 | -0.03 | 3.32 | 3.36 | -0.04 | 3.40 | 3.36 | | +0.04 |
| 3 | 5.53 | 5.48 | +0.05 | 5.60 | 5.67 | -0.07 | 5.64 | 5.57 | | +0.07 |
| 4 | - | - | - | - | - | - | - | - | | - |
| 5 | 5.75 | 5.69 | +0.06 | 5.17 | 5.20 | -0.03 | 5.31 | 5.26 | | +0.04 |
| 6 | 2.04 | 2.00 | +0.04 | 1.68 | 1.64 | +0.04 | 1.70 | 1.74 | | -0.04 |
| 7 | 0.95 | 0.93 | +0.02 | 0.90 | 0.82 | +0.08 | 0.83 | 0.89 | | -0.06 |
| 8 | 1.01 | 1.02 | -0.01 | 1.25 | 1.30 | -0.05 | 1.26 | 1.25 | | +0.01 |
| 9 | 1.58 | 1.41 | +0.17 | 1.54 | 1.69 | -0.15 | 1.70 | 1.53 | | +0.17 |
| 1’ | - | - | - | - | - | - | - | - | | - |
| 2’ | 2.96 | 3.00 | -0.04 | 3.42 | 3.46 | -0.04 | 3.46 | 3.45 | | +0.01 |
| 3’ | 4.21 | 4.20 | +0.01 | 4.29 | 4.28 | +0.01 | 4.39 | 4.38 | | +0.01 |
| 4’ | 3.46 | 3.42 | +0.04 | 3.02 | 3.00 | +0.02 | 3.07 | 3.05 | | +0.02 |
| 5’ | - | - | - | - | - | - | - | - | | - |
| 6’ | 4.11  3.78 | 4.26  4.01 | -0.15  -0.23 | n.d.  n.d. | n.d.  n.d. | -  - | n.d.  n.d. | n.d.  n.d. | | -  - |
| 7’ | 1.27 | 1.22 | +0.05 | 1.37 | 1.36 | +0.01 | 1.38 | 1.37 | | +0.01 |
| 8’ | 1.37 | 1.35 | +0.02 | 1.29 | 1.27 | +0.02 | 1.30 | 1.30 | | 0 |

n.d.: not detected due to overlap with MTPA signals.

## Supplementary Figures


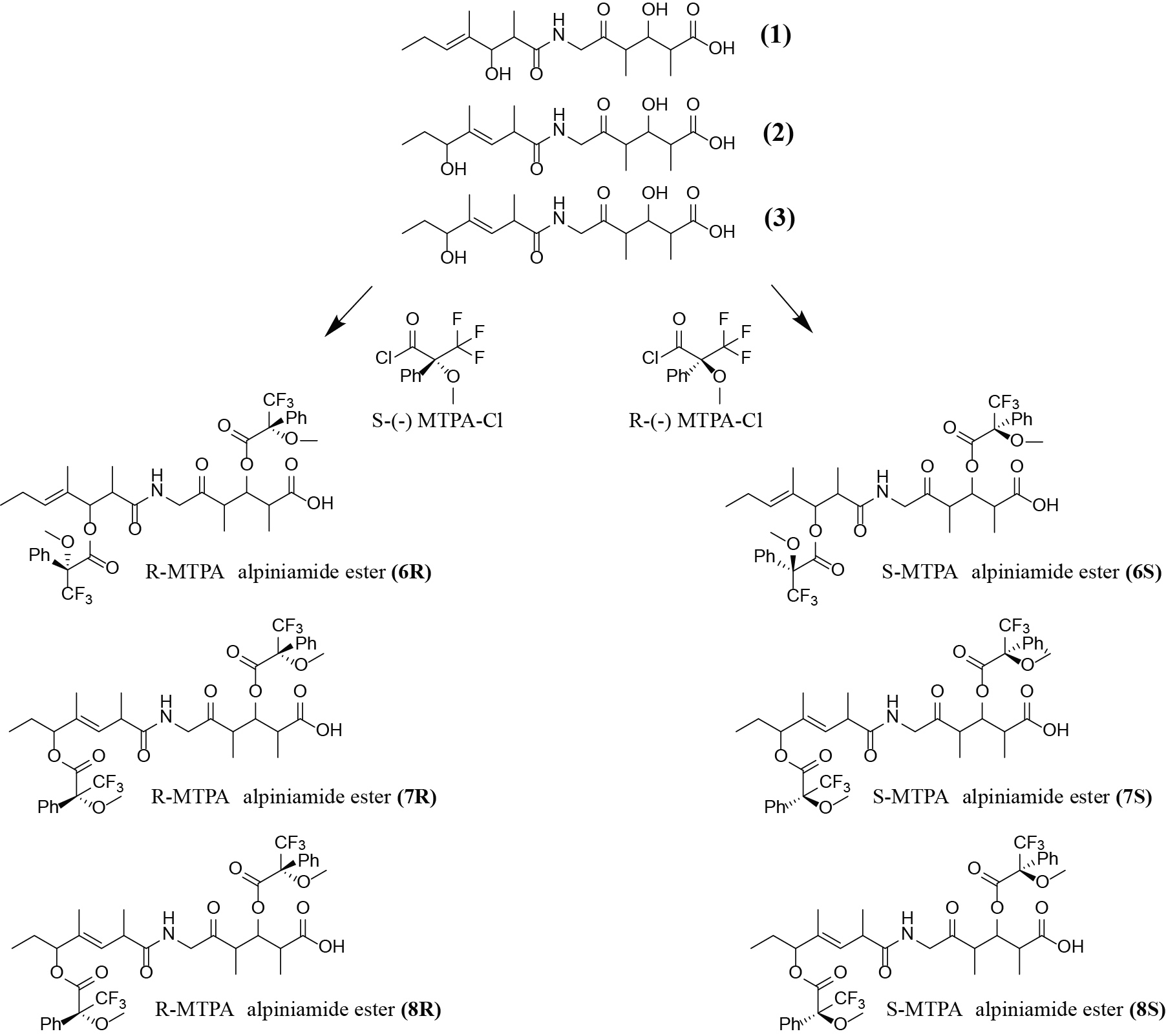


**Supplementary figure S1.** Reaction of alpiniamides with R-MTPA-Cl and S-MTPA-Cl in order to determine the absolute configuration.


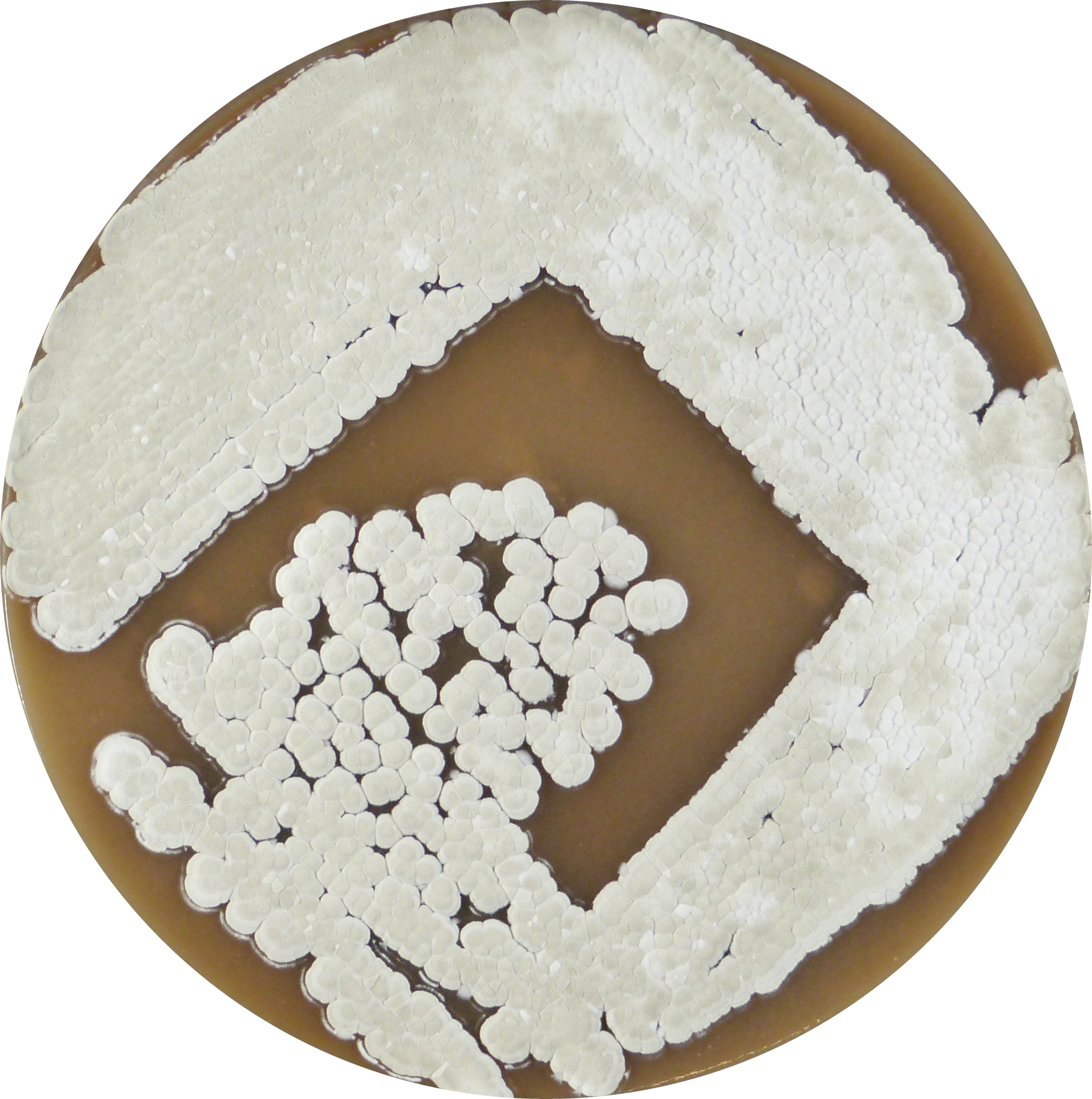


**Supplementary figure S2.** *Streptomycetes sp.* IB2014/011-12 grown on MS medium for 7 days.


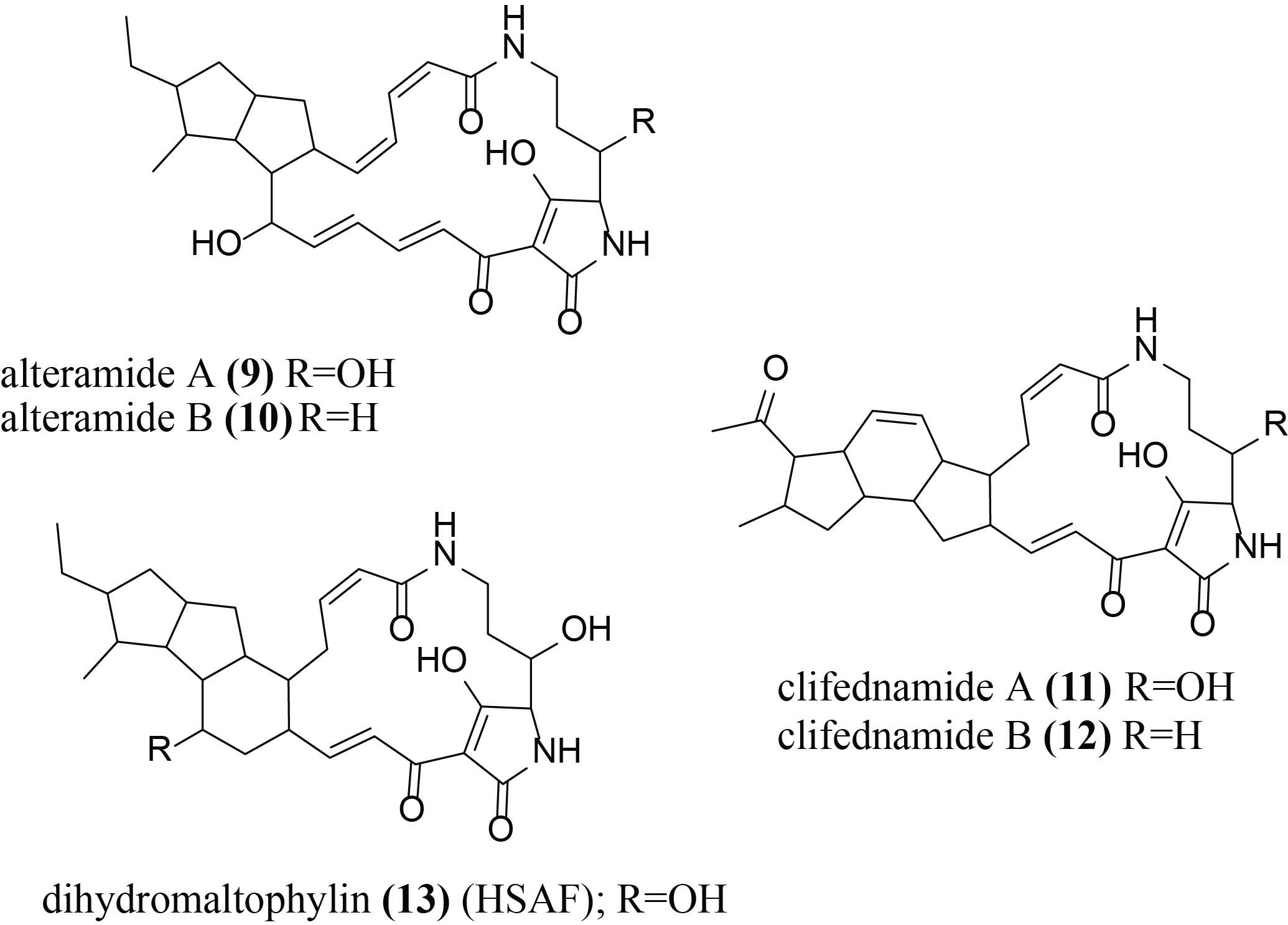


**Supplementary figure S3.** Structures of polycyclic tetramate macrolactams **(9-13)** identified in the extract of *Streptomyces sp.* IB2014/011-12.


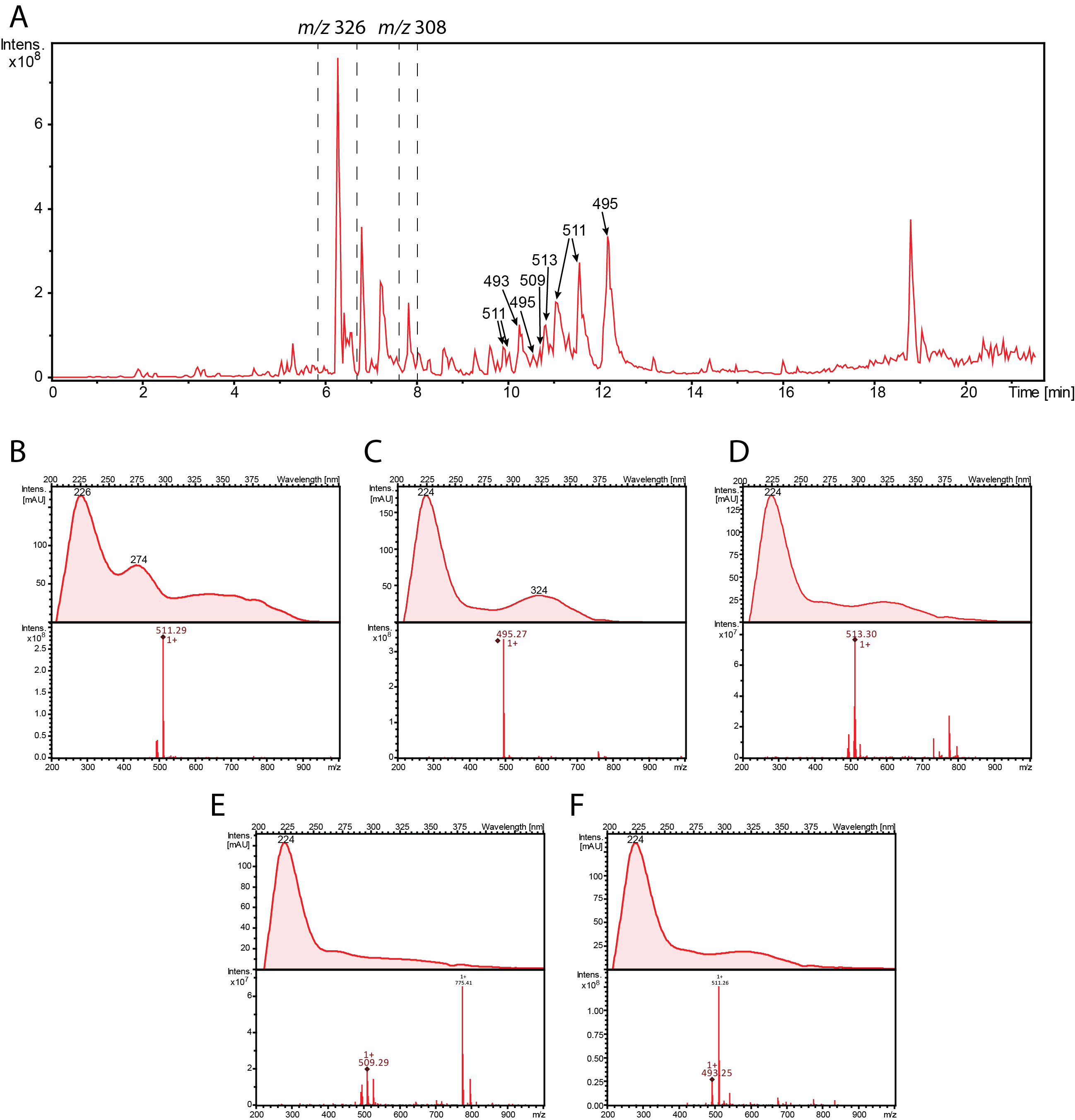


**Supplementary figure S4.** **(A)** MS and UV spectra of polycyclic tetramate macrolactams identified in the extract of *Streptomycetes sp.* IB2014/011-12. **(B)** alteramide A **(9)**; **(C)** alteramide B **(10)**; **(D)** clifednamide A **(11)**; **(E)** clifednamide B **(12)**; **(F)** dihydromaltophylin **(13)** (HSAF).


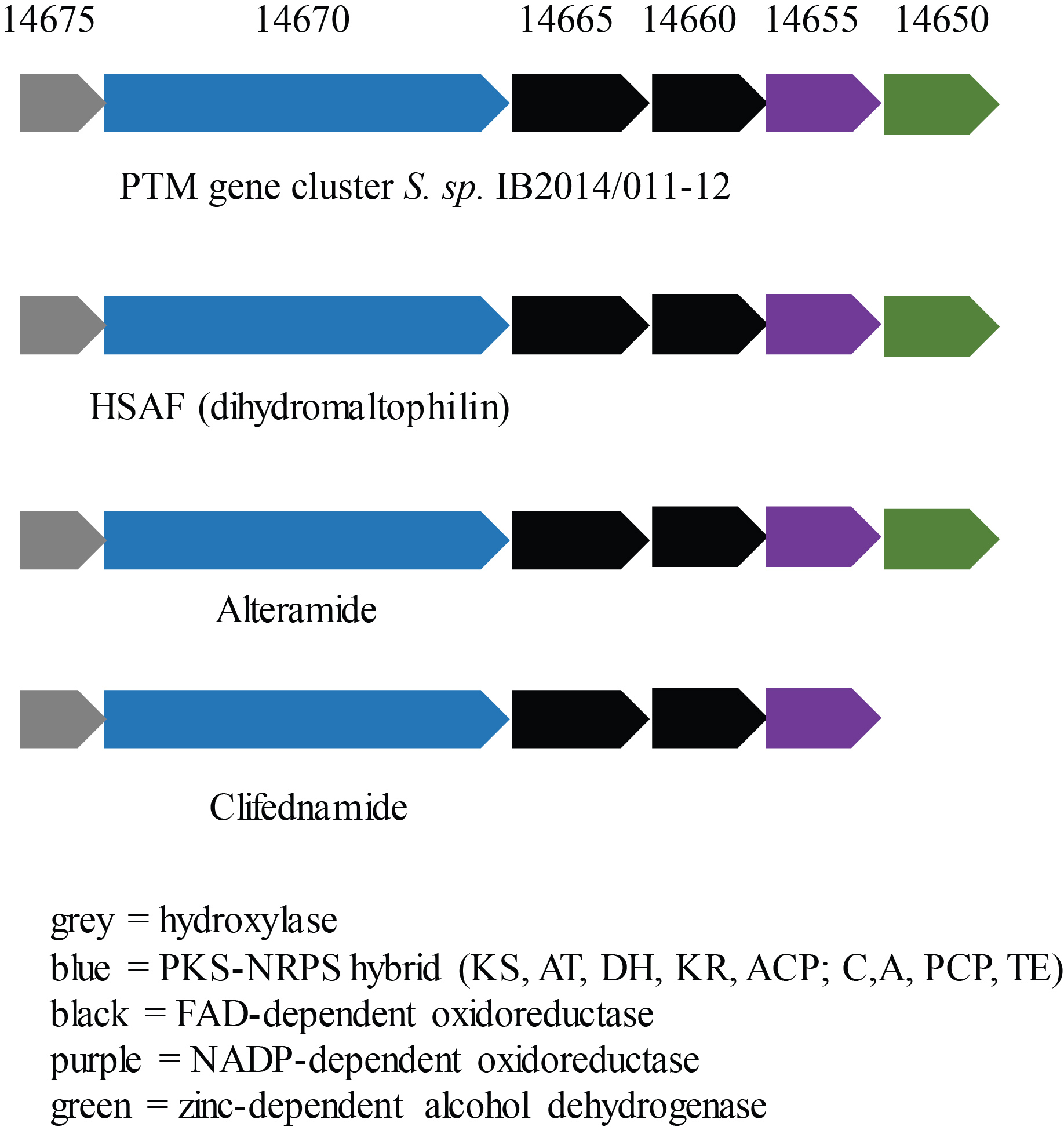


**Supplementary figure S5.** Genetic organization of gene cluster for PTMs production from the genome of *Streptomyces sp.* IB2014/011-12 and gene cluster of dihydromaltophylin, alteramide and clifednamide.


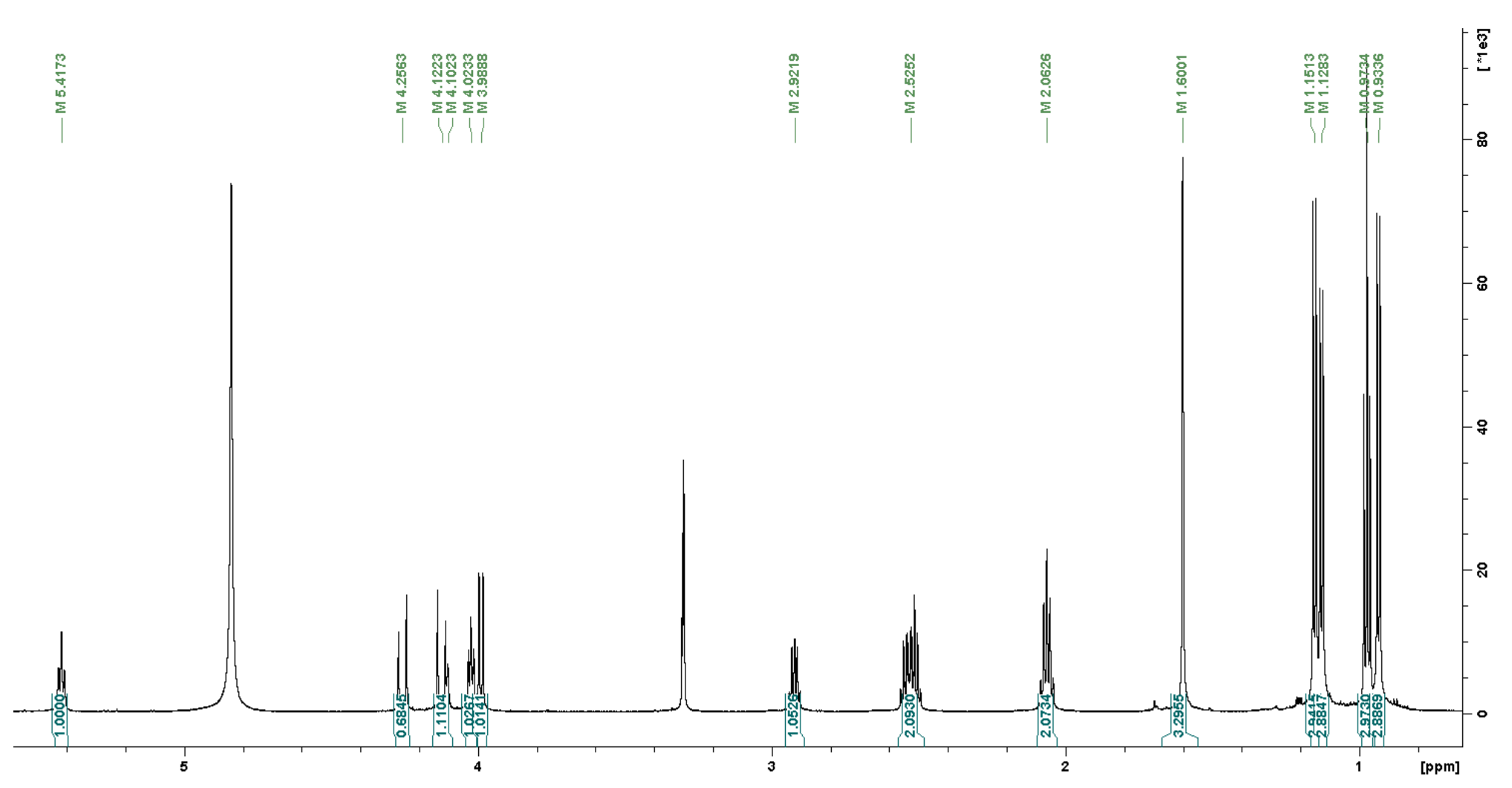


**Supplementary figure S6.** ^1^H NMR-spectra of alpiniamide A in MeOD_4_ 500 MHz.


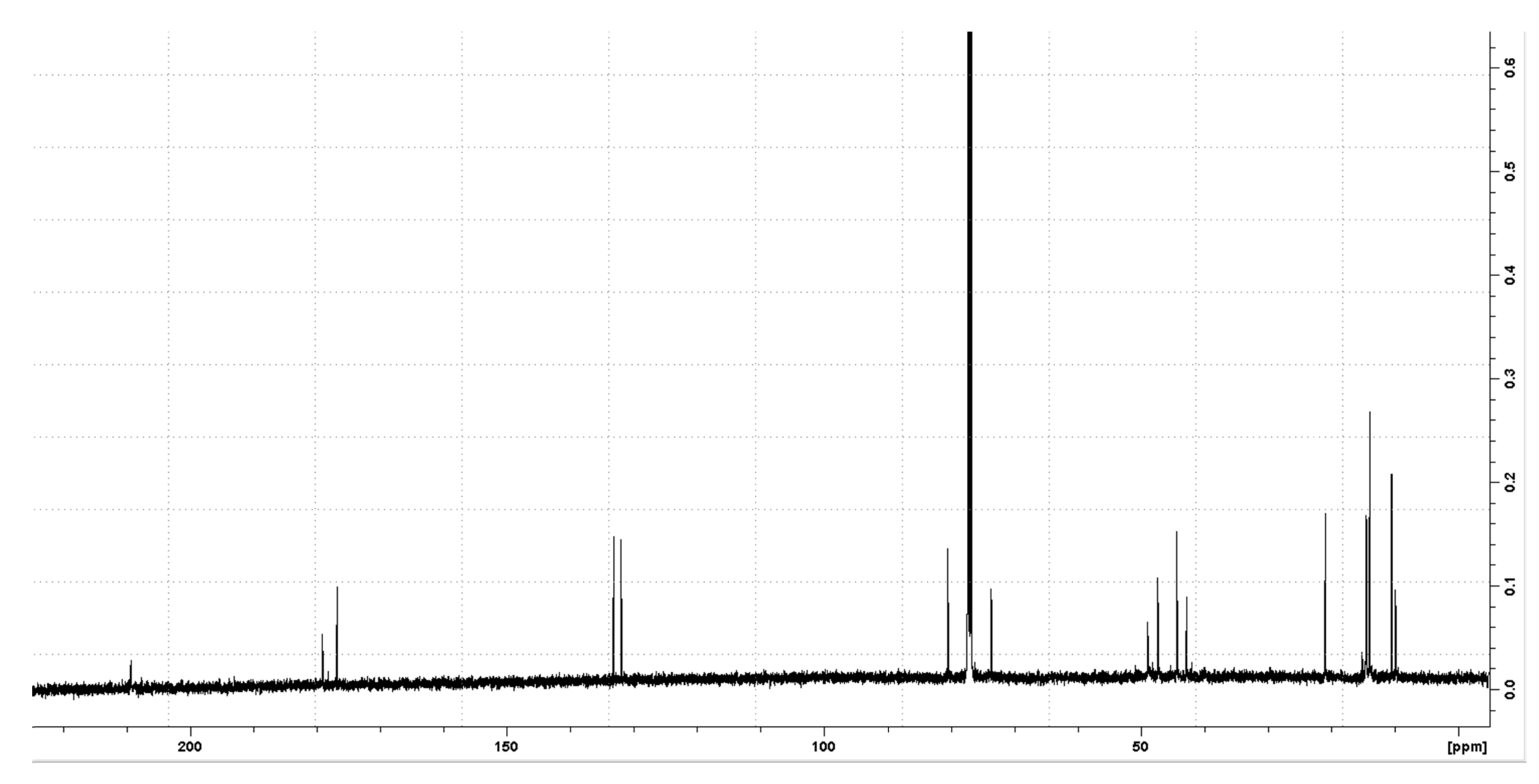


**Supplementary figure S7.** ^13^C NMR-spectra of alpiniamide A in CDCl_3_ 500 MHz.

**
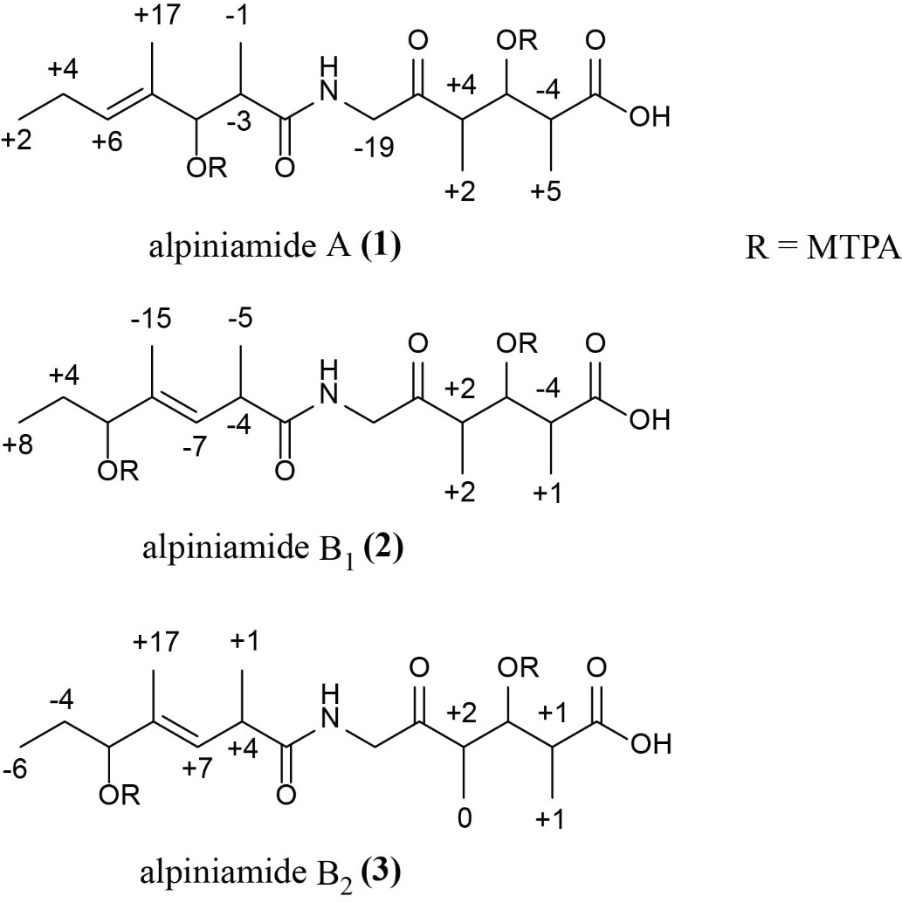
**

**Supplementary figure S8.** ∆δ_(S-R)_ values (x10^-2^ ppm) of the MTPA esters of **1-3**.


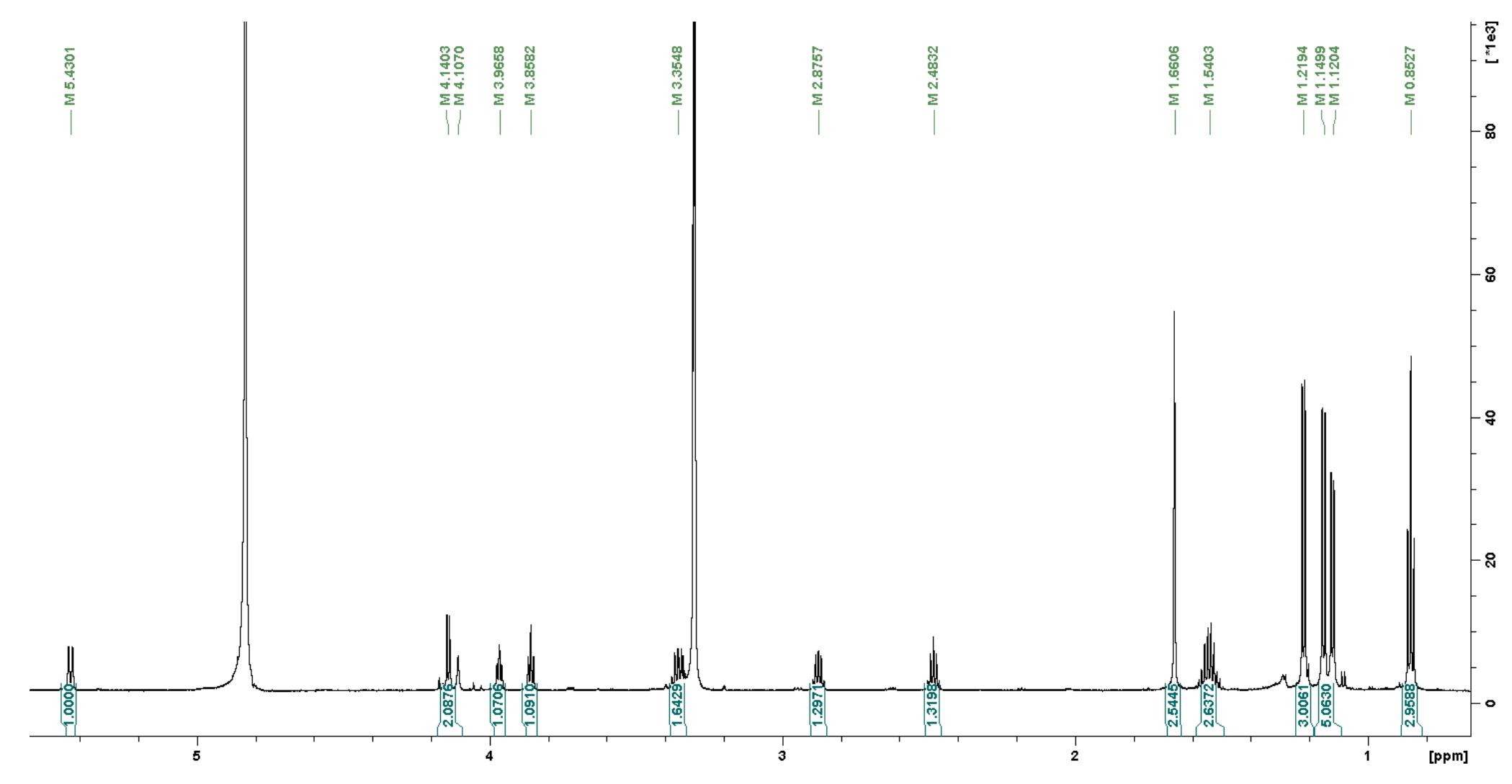


**Supplementary figure S9.** ^1^H NMR-spectra of alpiniamide B_1_ in MeOD_4_ 500 MHz.


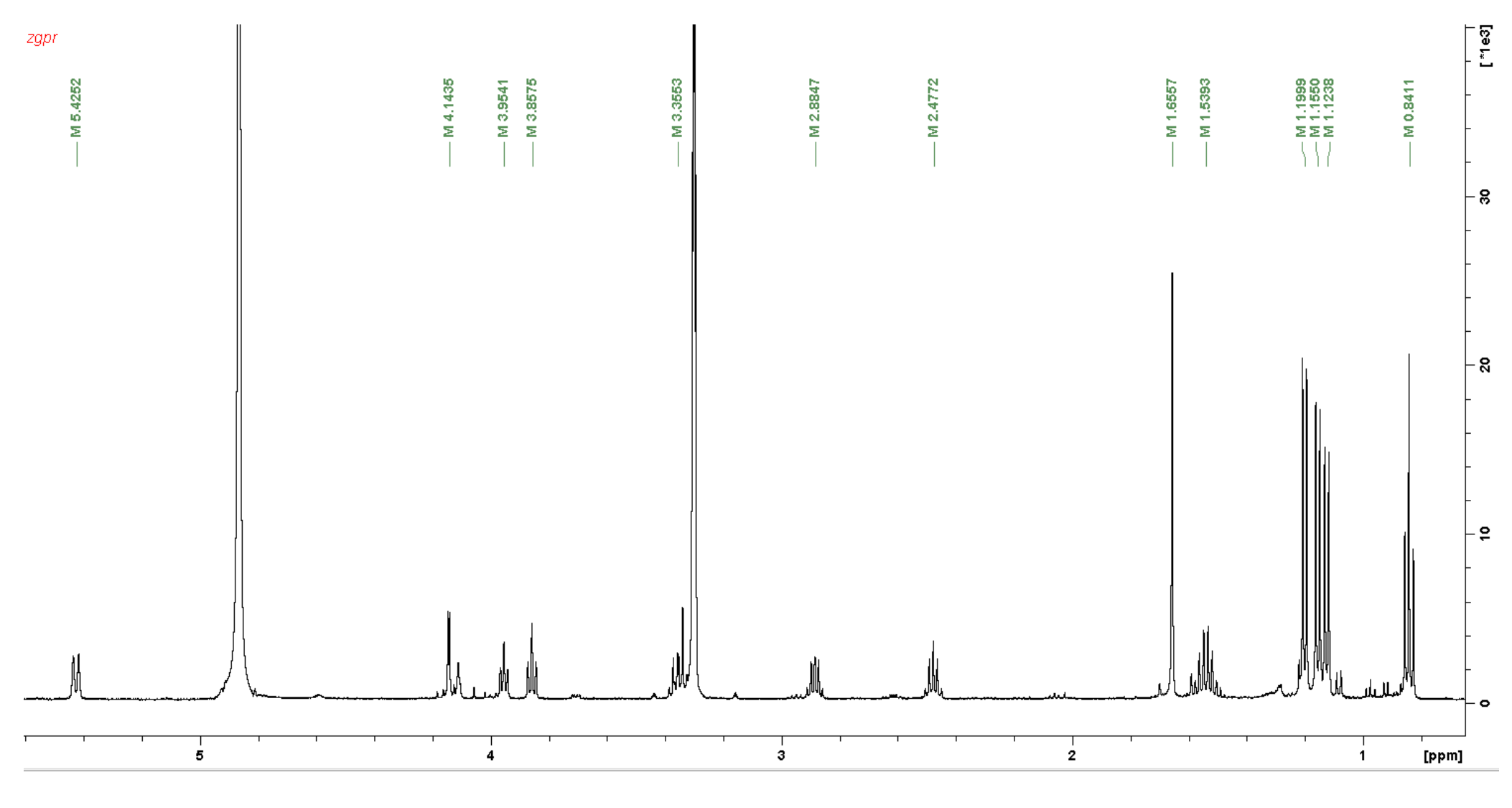


**Supplementary figure S10.** ^1^H NMR-spectra of alpiniamide B_2_ in MeOD_4_ 500 MHz.


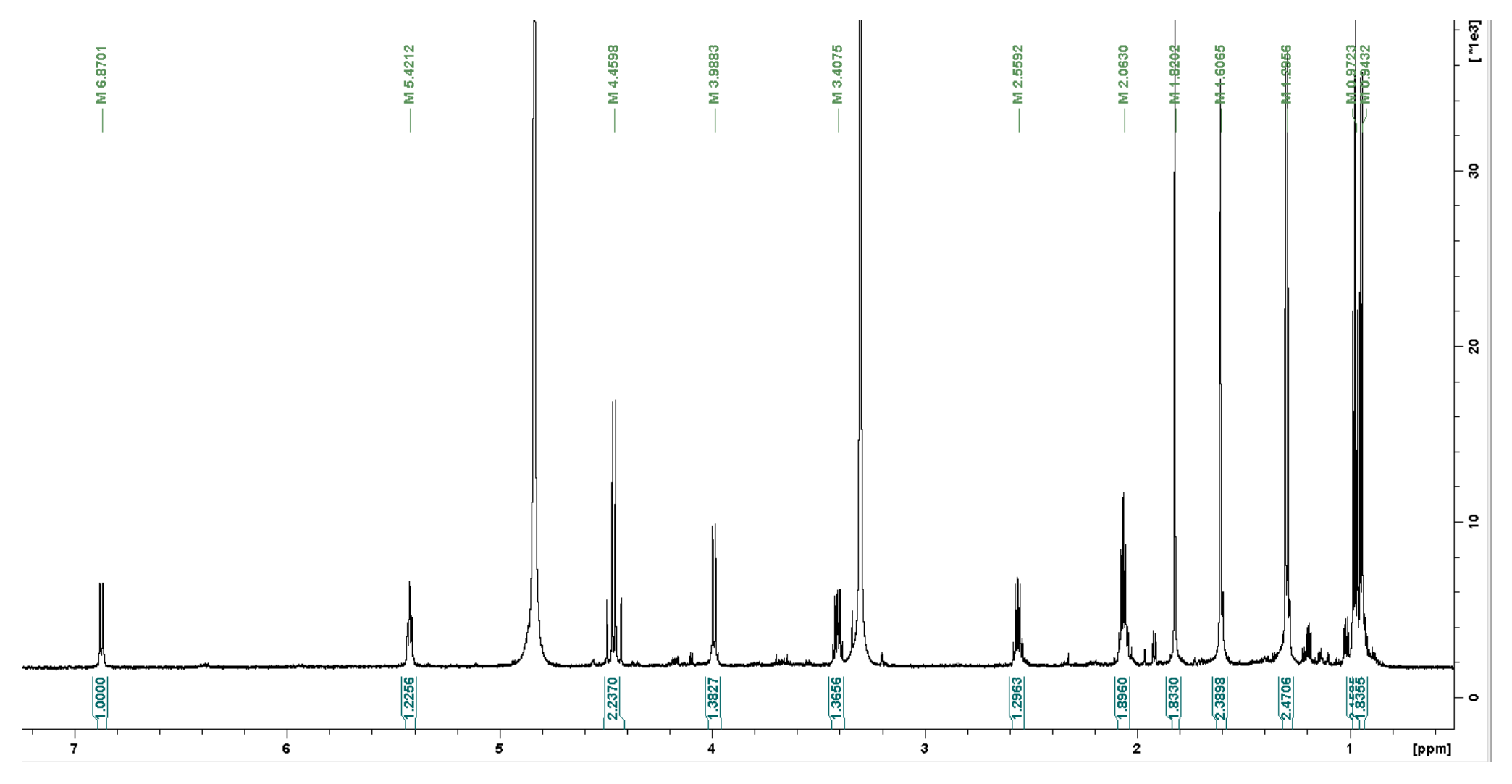


**Supplementary figure S11.** ^1^H NMR-spectra of alpiniamide C in MeOD_4_ 500 MHz.


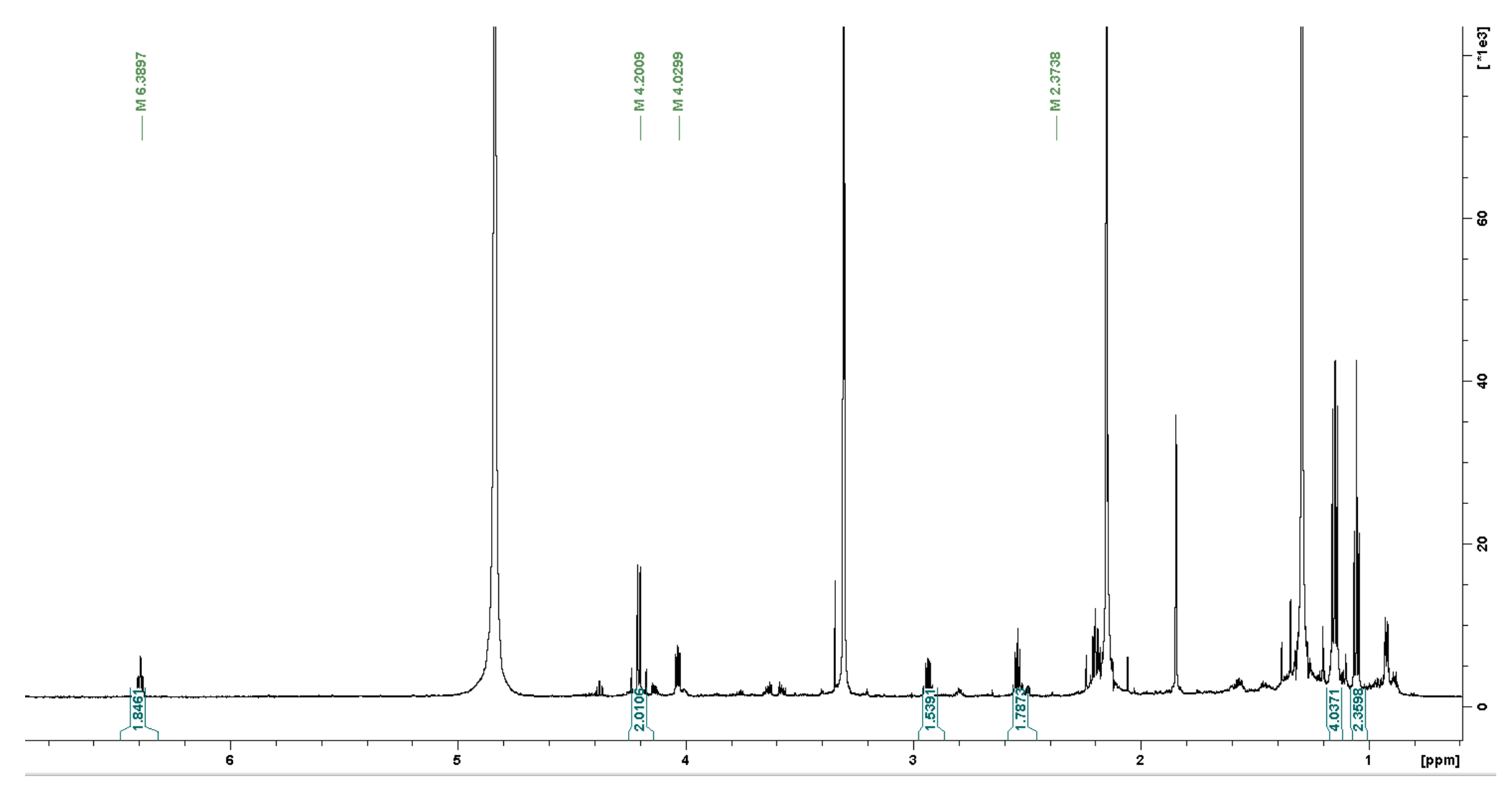


**Supplementary figure S12.** ^1^H NMR-spectra of alpiniamide D in MeOD_4_ 500 MHz.


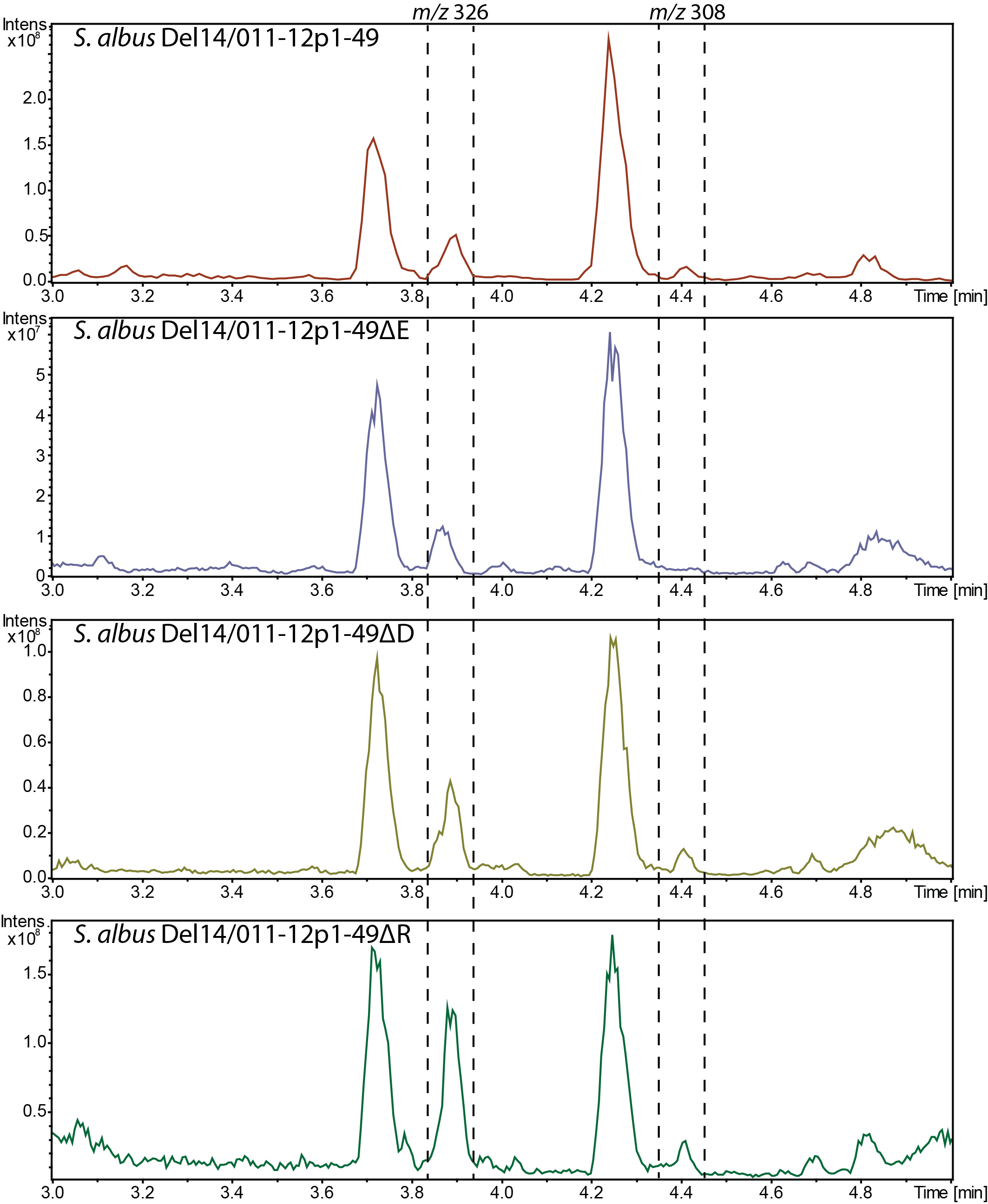


**Supplementary figure S13.** LC-MS chromatogram of extracts of *S. albus* Del14 carrying 011-12p1-49 plasmid and it’s variants with the deletion of individual *alp* genes: *S. albus* Del14/011-12p1-49∆E – *alpE*; *S. albus* Del14/011-12p1-49∆D – *alpD; S. albus* Del14/011-12p1-49∆R – *alpR.* Peak that corresponds to alpiniamides are highlighted: *m/z* 326 [*M*-H_2_O+H]^+^ for alpiniamides A and B, m/z 308 [*M*-H_2_O+H]^+^ for alpiniamide C. In the case of *S. albus* Del14/011-12p1-49∆E small peak eluting around same RT as alpiniamide A has *m/z* of 285 [*M*+H]^+^.


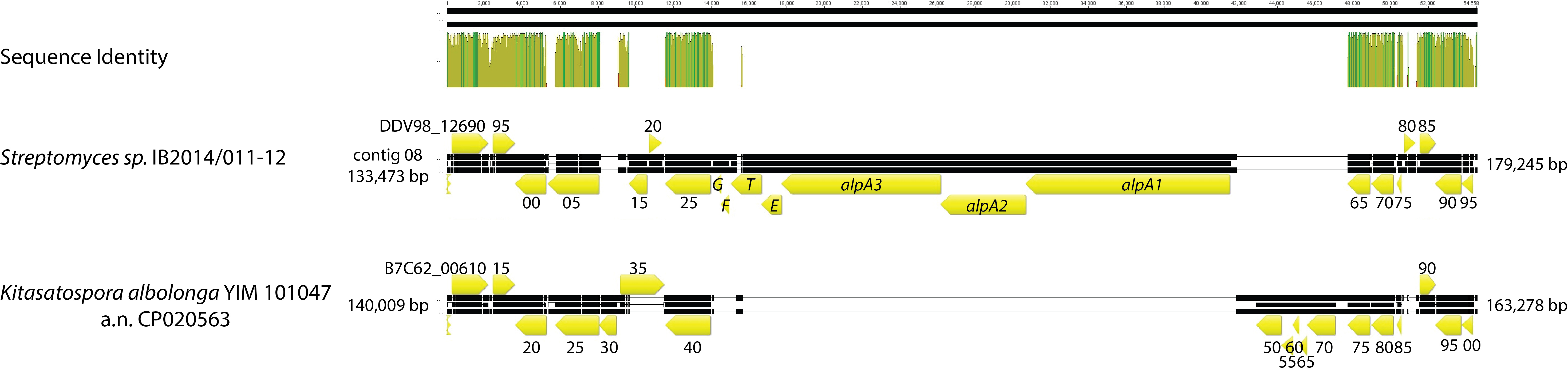


**Supplementary figure S14.** Alignment of whole genome of *Kitasatospora albolonga* YIM 101047 (GenBank accession number CP020563) and contig 08 of *Streptomyces sp.* IB2014/011-12 genome containing *alp* gene cluster and regions surrounding it. The alignment was performed with Geneious v. 8.1.7 software (Biomatters Ltd., New Zealand) using progressive Mauve algorithm.

| 10 20 30 40 50 60 70 80  ....*....\|....*....\|....*....\|....*....\|....*....\|....*....\|....*....\|....*....\|  [gi 17548863](https://www.ncbi.nlm.nih.gov/entrez/query.fcgi?cmd=Search&doptcmdl=GenPept&db=Protein&term=17548863) 5391 RPLFLVHEITGLDGYFTQLGAWIDAD-IPVYGLPAVGWG----EPQLRTIEGLAKRLKAAMRAVQPHG---PYRLAGWSF 5462  query 1 RPVFWIHGALAGVESYRTIAERI--D-RPFYGIQARGLLt--eDEPIEGITAMAEHYTEVIRSVQPEG---PYDVGGFCL 72  [gi 17989334](https://www.ncbi.nlm.nih.gov/entrez/query.fcgi?cmd=Search&doptcmdl=GenPept&db=Protein&term=17989334) 1 -------------------MGLLAGAaLVLPSLPAEAKt-----QQAAMPPNATSPHQADVYLLRGFA---DIFSTGIDE 53  [gi 730829](https://www.ncbi.nlm.nih.gov/entrez/query.fcgi?cmd=Search&doptcmdl=GenPept&db=Protein&term=730829) 1058 QIIFAFPPVLGYG-------LMYQN-----LSSRLPSYKl--cAFDFIEEEDRLDRYADLIQKLQPEG---PLTLFGY**S**A 1120  [gi 19554061](https://www.ncbi.nlm.nih.gov/entrez/query.fcgi?cmd=Search&doptcmdl=GenPept&db=Protein&term=19554061) 1348 PAVFMFHPAGGS-------SVVYQPL-MRRLPEDVPVYg---vERLEGDLADRAAAYVDDIKKYSDGF---PVVLGGWSF 1413  [gi 15610936](https://www.ncbi.nlm.nih.gov/entrez/query.fcgi?cmd=Search&doptcmdl=GenPept&db=Protein&term=15610936) 1469 VPVFVFHPAGGS-------TVVYEPL-LGRLPADTPMYg---fERVEGSIEERAQQYVPKLIEMQGDG---PYVLVGWSL 1534  [gi 2120647](https://www.ncbi.nlm.nih.gov/entrez/query.fcgi?cmd=Search&doptcmdl=GenPept&db=Protein&term=2120647) 2170 SPLFMFHPSFGSVHCYKTLAMALRDR-HPVKGVVCRALLg--aGREVPEWDDMVAEYAEQLLQEHPEG---VFNLAGWSL 2243  [gi 15598523](https://www.ncbi.nlm.nih.gov/entrez/query.fcgi?cmd=Search&doptcmdl=GenPept&db=Protein&term=15598523) 2077 PPLFLVHPLGGHVLCYLPLVRALPPD-QPVYALQAAGTGq--gSTPLAVLEDIAASYLAAIRRVQPEG---PYYLGGWSF 2150  [gi 17546530](https://www.ncbi.nlm.nih.gov/entrez/query.fcgi?cmd=Search&doptcmdl=GenPept&db=Protein&term=17546530) 1727 RTLVCVHASDGHAAAYRPLAAVLAGT-VQCVALQSPGLEa--gQAPLRSVEAQAACYLAALRAGREAGaqaPWHVLGWSM 1803  [gi 17549641](https://www.ncbi.nlm.nih.gov/entrez/query.fcgi?cmd=Search&doptcmdl=GenPept&db=Protein&term=17549641) 1173 PAVFAIPGVGGLAAAFAPLGTELRRHgIELVAFDSPGWHadrtLPIDARLLAYVQHVVDAIRRRTPHG---PYQLIGHSF 1249 |
| --- |
| 90 100 110 120 130 140 150 160  ....*....\|....*....\|....*....\|....*....\|....*....\|....*....\|....*....\|....*....\|  [gi 17548863](https://www.ncbi.nlm.nih.gov/entrez/query.fcgi?cmd=Search&doptcmdl=GenPept&db=Protein&term=17548863) 5463 GGVLAYEIAIQLIGED-EEVEFLGLLDTR-------QPalvsGGKPKWAAENRPH----HAQLLELCLAYWQ-QRSPGgP 5529  query 73 GGIVAYEVTRRLQAQG-QDVASLTMVDSPdetglakSN----ANGFQSARSAALQvvnsLLWPAGEKDPAALrARLVH-R 146  [gi 17989334](https://www.ncbi.nlm.nih.gov/entrez/query.fcgi?cmd=Search&doptcmdl=GenPept&db=Protein&term=17989334) 54 IGAELQAAGVNAHVQG-HAAWRLVLNRIV-------AD----------------------------QQKNGH-LPVVL-- 94  [gi 730829](https://www.ncbi.nlm.nih.gov/entrez/query.fcgi?cmd=Search&doptcmdl=GenPept&db=Protein&term=730829) 1121 GCSLAFEAAKKLEEQG-RIVQRIIMV**D**SY-------KK---------QGVSD--------------LDGRTV-ESDV--- 1165  [gi 19554061](https://www.ncbi.nlm.nih.gov/entrez/query.fcgi?cmd=Search&doptcmdl=GenPept&db=Protein&term=19554061) 1414 GGAVAFEVAHQLVGSD-VEVATVALLDTV-------QPsn-pAPDTAEETRARWT----RYADFAKKTY-GL-DFEVP-F 1477  [gi 15610936](https://www.ncbi.nlm.nih.gov/entrez/query.fcgi?cmd=Search&doptcmdl=GenPept&db=Protein&term=15610936) 1535 GGVLAYACAIGLRRLG-KDVRFVGLIDAV-------RAge-eIPQTKEEIRKRWD----RYAAFAEKTFNVT-IPAIP-Y 1599  [gi 2120647](https://www.ncbi.nlm.nih.gov/entrez/query.fcgi?cmd=Search&doptcmdl=GenPept&db=Protein&term=2120647) 2244 GGNLAMDVAARLEQRG-RQVAFVGWIDAP-------AP---------VRVEAFWN----EIGPTPEAVPNLS-VGEMR-V 2300  [gi 15598523](https://www.ncbi.nlm.nih.gov/entrez/query.fcgi?cmd=Search&doptcmdl=GenPept&db=Protein&term=15598523) 2151 GGFVAYEMARQLRALDpQAVAQLIVLDSI-------TVdr-nHAG--SASDEALL----LFFYWELVWFERS-DKEVEpL 2215  [gi 17546530](https://www.ncbi.nlm.nih.gov/entrez/query.fcgi?cmd=Search&doptcmdl=GenPept&db=Protein&term=17546530) 1804 GAYVAVEMARQLAQAG-ECVAQLLLVDPA-------PQ----EAMRAAARSEYDL----LLSLAPEAVRREL-AEQVGsA 1866  [gi 17549641](https://www.ncbi.nlm.nih.gov/entrez/query.fcgi?cmd=Search&doptcmdl=GenPept&db=Protein&term=17549641) 1250 GARVAFDVALALEEAG--GTVALTMLDAL-------PG------N--DLVDMSRW----RVGQTPRELAGWL-LGAMQ-- 1305 |
| 170 180 190 200 210 220 230 240  ....*....\|....*....\|....*....\|....*....\|....*....\|....*....\|....*....\|....*....\|  [gi 17548863](https://www.ncbi.nlm.nih.gov/entrez/query.fcgi?cmd=Search&doptcmdl=GenPept&db=Protein&term=17548863) 5530 ESAKLAGLAGVEDFSALLERCRAQallAPDL-ADVTEPDLW--HVLDRIVAHGDAQANYTVfpmplKLHLFVAAHEQRDD 5606  query 147 DEIADDLDEDAFVL-RLAELAAER---GLAMrPDRTARFVRrnMAIQLAYRLGEHTIRPLP-----RPEAVVctyfrnrr 217  [gi 17989334](https://www.ncbi.nlm.nih.gov/entrez/query.fcgi?cmd=Search&doptcmdl=GenPept&db=Protein&term=17989334) 95 -IGHSLGANAAIYIAEELERRGIA---VDYM-ATFAATGPD--PLPGNVRRVVNFYFKQHG-----WGLPLVPGPR---- 158  [gi 730829](https://www.ncbi.nlm.nih.gov/entrez/query.fcgi?cmd=Search&doptcmdl=GenPept&db=Protein&term=730829) 1166 EALMNVNRDN---EALNSEAVKHG---LKQK-THAFYSYYV--NLISTGQVKADIDLLTSG--------ADFDMPE---- 1224  [gi 19554061](https://www.ncbi.nlm.nih.gov/entrez/query.fcgi?cmd=Search&doptcmdl=GenPept&db=Protein&term=19554061) 1478 EILDTIGEDG--MLSMMTDFLANTdasEHGL-SAGVLEHQR--ASFVDNRILAKLNFADWAnv--eAPVILFRAERMHDG 1550  [gi 15610936](https://www.ncbi.nlm.nih.gov/entrez/query.fcgi?cmd=Search&doptcmdl=GenPept&db=Protein&term=15610936) 1600 EQLEELDDEG--QVRFVLDAVSQS---GVQI-PAGIIEHQR--TSYLDNRAIDTAQIQPYD-----GHVTLYMADRYHDD 1666  [gi 2120647](https://www.ncbi.nlm.nih.gov/entrez/query.fcgi?cmd=Search&doptcmdl=GenPept&db=Protein&term=2120647) 2301 ELLGVMFPERAEHIERAWSSICSAttdDEQR-WTRMSDWAE--AEIGAEFATLRSEIAQSN-----ELEVSWELKQILDE 2372  [gi 15598523](https://www.ncbi.nlm.nih.gov/entrez/query.fcgi?cmd=Search&doptcmdl=GenPept&db=Protein&term=15598523) 2216 PEGASLEQKLDHIVERAIEAGVLPagtPRAT-VQRLYELFR--ASWQALIGYRPEVSDQDMtllraDGPLPLALKPMHDA 2292  [gi 17546530](https://www.ncbi.nlm.nih.gov/entrez/query.fcgi?cmd=Search&doptcmdl=GenPept&db=Protein&term=17546530) 1867 DAFASLPPARRLAHWRAGLRLAAPs--PADD-DAALERMVA--VLLANVTAMVDYRLPILD-----LPTVALYQASEHPA 1936  [gi 17549641](https://www.ncbi.nlm.nih.gov/entrez/query.fcgi?cmd=Search&doptcmdl=GenPept&db=Protein&term=17549641) 1306 --NGDVPAEPGEDAVASLARLQIYg--DLDV-HDALALVDD--QMLASRRYRPARRLRATR------VQMVYAEHGLIGA 1372 |
| 250 260 270 280 290 300  ....*....\|....*....\|....*....\|....*....\|....*....\|....*....\|....*..  [gi 17548863](https://www.ncbi.nlm.nih.gov/entrez/query.fcgi?cmd=Search&doptcmdl=GenPept&db=Protein&term=17548863) 5607 EPP---------------PHKRWLGWNAILPDTQlqrivV--PGTHQSMVLE-HAQALGEALSAALHAAAGQPQP 5663  query 218 glflgevepyfqvtgetfsldhvnyrqdwgrempglrlveidaanHmtilndaep-------------------- 272  [gi 17989334](https://www.ncbi.nlm.nih.gov/entrez/query.fcgi?cmd=Search&doptcmdl=GenPept&db=Protein&term=17989334) 159 --------------------FHGHLENRDFSNAK--------DVGHFNIEKQrPLQAEVVRDVLAVVNAD----- 200  [gi 730829](https://www.ncbi.nlm.nih.gov/entrez/query.fcgi?cmd=Search&doptcmdl=GenPept&db=Protein&term=730829) 1225 ------------------WLASWEEATTGVYRVKr------GFGT**H**AEMLQG-ETLDRNAEILLEFLNTQTVTVS 1274  [gi 19554061](https://www.ncbi.nlm.nih.gov/entrez/query.fcgi?cmd=Search&doptcmdl=GenPept&db=Protein&term=19554061) 1551 AIElepnyakidQDGGWSGIVNDLEIVQ-------------LNGDHLAVVDE-PEIGTVGAHLSRRIDEISRKN- 1610  [gi 15610936](https://www.ncbi.nlm.nih.gov/entrez/query.fcgi?cmd=Search&doptcmdl=GenPept&db=Protein&term=15610936) 1667 AIMfepryavrqPDGGWGEYVSDLEVVP-------------IGGEHIQAIDE-PIIAKVGEHMSRALGQIEADRT 1727  [gi 2120647](https://www.ncbi.nlm.nih.gov/entrez/query.fcgi?cmd=Search&doptcmdl=GenPept&db=Protein&term=2120647) 2373 RLKamdyprltaKVSLWWAARSTNAIQRs-----------aVERSMAEAIGA-ERVEPVRVLDTRHDKIIDHPEF 2435  [gi 15598523](https://www.ncbi.nlm.nih.gov/entrez/query.fcgi?cmd=Search&doptcmdl=GenPept&db=Protein&term=15598523) 2293 AGThy-----gdPKNGWQHWTSGRLDVId------------VPGDHLVLMKE-PYVETVAAEIAALLEPSTSSER 2349  [gi 17546530](https://www.ncbi.nlm.nih.gov/entrez/query.fcgi?cmd=Search&doptcmdl=GenPept&db=Protein&term=17546530) 1937 GWGd--------VIAPWRDVFPRGIQAEt------------LAGTHWSVVGAeVLGPVLAQRLREGGAAAPATPA 1991  [gi 17549641](https://www.ncbi.nlm.nih.gov/entrez/query.fcgi?cmd=Search&doptcmdl=GenPept&db=Protein&term=17549641) 1373 HAHd-------aIVDSLRAWADTVAVAR-------------LDADHFSMLKA-APALAGHVMAHGRA-------- 1418 |

**Supplementary figure S15**. Multiple sequence alignment of TE domain of AlpA2 using the Conserved Domain BLAST with the closest representatives of COG3319 super-family. The proposed active site residues (catalytic triad) are marked with red frames. The catalytic triad is deduced based on structural data for thioeserase domain of surfactin synthetase subunit 3 SrfA-C (730829, highlighted in yellow) (Bruner et al., 2002).

REFERENCES

Barona-Gómez, F., Wong, U., Giannakopulos, A. E., Derrick, P. J., and Challis, G. L. (2004). Identification of a cluster of genes that directs desferrioxamine biosynthesis in Streptomyces coelicolor M145. *Journal of the American Chemical Society* 126, 16282–16283. doi: 10.1021/ja045774k

Bruner, S. D., Weber, T., Kohli, R. M., Schwarzer, D., Marahiel, M. A., Walsh, C. T., et al. (2002). Structural basis for the cyclization of the lipopeptide antibiotic surfactin by the thioesterase domain SrfTE. *Structure (London, England : 1993)* 10, 301–310.

Challis, G. L., and Ravel, J. (2000). Coelichelin, a new peptide siderophore encoded by the Streptomyces coelicolor genome: Structure prediction from the sequence of its non-ribosomal peptide synthetase. *FEMS Microbiology Letters* 187, 111–114. doi: 10.1111/j.1574-6968.2000.tb09145.x

Cook, D., Rimando, A. M., Clemente, T. E., Schröder, J., Dayan, F. E., Nanayakkara, N. P. D., et al. (2010). Alkylresorcinol synthases expressed in Sorghum bicolor root hairs play an essential role in the biosynthesis of the allelopathic benzoquinone sorgoleone. *The Plant cell* 22, 867–887. doi: 10.1105/tpc.109.072397.

Kersten, R. D., Yang, Y.-L., Xu, Y., Cimermancic, P., Nam, S.-J., Fenical, W., et al. (2011). A mass spectrometry-guided genome mining approach for natural product peptidogenomics. *Nature chemical biology* 7, 794–802. doi: 10.1038/nchembio.684.

Luo, Y., Huang, H., Liang, J., Wang, M., Lu, L., Shao, Z., et al. (2013). Activation and characterization of a cryptic polycyclic tetramate macrolactam biosynthetic gene cluster. *Nature communications* 4, 2894. doi: 10.1038/ncomms3894

Pan, J.-J., Solbiati, J. O., Ramamoorthy, G., Hillerich, B. S., Seidel, R. D., Cronan, J. E., et al. (2015). Biosynthesis of Squalene from Farnesyl Diphosphate in Bacteria: Three Steps Catalyzed by Three Enzymes. *ACS central science* 1, 77–82. doi: 10.1021/acscentsci.5b00115

Schwarz, J., Konjik, V., Jankowitsch, F., Sandhoff, R., and Mack, M. (2016). Identification of the Key Enzyme of Roseoflavin Biosynthesis. *Angewandte Chemie (International ed. in English)* 55, 6103–6106. doi: 10.1002/anie.201600581

Suzuki, H., Ohnishi, Y., and Horinouchi, S. (2007). GriC and GriD constitute a carboxylic acid reductase involved in grixazone biosynthesis in Streptomyces griseus. *J. Antibiot.* 60, 380–387. doi: 10.1038/ja.2007.52

Ueda, K., Oinuma, K.-I., Ikeda, G., Hosono, K., Ohnishi, Y., Horinouchi, S., et al. (2002). AmfS, an Extracellular Peptidic Morphogen in Streptomyces griseus. *Journal of Bacteriology* 184, 1488–1492. doi: 10.1128/JB.184.5.1488–1492.2002

Woo, P. C. Y., Tam, E. W. T., Chong, K. T. K., Cai, J. J., Tung, E. T. K., Ngan, A. H. Y., et al. (2010). High diversity of polyketide synthase genes and the melanin biosynthesis gene cluster in Penicillium marneffei. *The FEBS journal* 277, 3750–3758. doi: 10.1111/j.1742-4658.2010.07776.x

Zhu, D., Liu, J., Han, R., Shen, G., Long, Q., Wei, X., et al. (2014). Identification and characterization of ectoine biosynthesis genes and heterologous expression of the ectABC gene cluster from Halomonas sp. QHL1, a moderately halophilic bacterium isolated from Qinghai Lake. *Journal of microbiology (Seoul, Korea)* 52, 139–147. doi: 10.1007/s12275-014-3389-5.
